# A biological condition gradient for Caribbean coral reefs: Part II. Numeric rules using sessile benthic organisms

**DOI:** 10.1016/j.ecolind.2022.108576

**Published:** 2022-02

**Authors:** Deborah L Santavy, Susan K Jackson, Benjamin Jessup, Christina Horstmann, Caroline Rogers, Ernesto Weil, Alina Szmant, David Cuevas Miranda, Brian K. Walker, Christopher Jeffrey, David Ballantine, William S Fisher, Randy Clark, Hector Ruiz Torres, Brandi Todd, Sandy Raimondo

**Affiliations:** a US Environmental Protection Agency (US EPA), Office of Research and Development (ORD), Center for Environmental, Measurement and Modeling (CEMM), Gulf Ecosystem Measurement and Modeling Division (GEMMD), Gulf Breeze, FL, United States; b US EPA, Office of Water (OW), Washington, DC, United States; c Tetra Tech, Inc., Montpelier, VT, United States; d Oak Ridge Institute for Science Education Participant at US EPA, ORD, CEMM, GEMMD, Gulf Breeze, FL, United States; e U.S. Geological Survey (USGS), Wetland and Aquatic Research Center, St. John, USVI, United States; f Department of Marine Sciences, University of Puerto Rico, Mayaguez, PR, United States; g University of North Carolina, Wilmington, NC, United States; h US EPA, Region 2, Caribbean Marine Protection Division, Guaynabo, PR, United States; i Nova Southeastern University Oceanographic Center, Dania, FL, United States; j CSS-Inc., Fairfax, VA, Under Contract to NOAA, National Centers for Coastal Ocean Science, Marine Spatial Ecology Division, Biogeography Branch, Silver Spring, MD, United States; k Smithsonian Institution, National Museum of Natural History, Wash, DC, United States; l NOAA NCCOS, Marine Spatial Ecology Division, Biogeography Branch, Stennis Space Center, MS, United States; m University of Puerto Rico, Rio Piedras, PR, United States; n NOAA, Emergency Response Division, New Orleans, LA, United States

**Keywords:** Coral reef condition, Biocriteria, Biological integrity, Biological Condition Gradient (BCG), Coral reef protection, numeric model

## Abstract

The Biological Condition Gradient (BCG) is a conceptual model used to describe incremental changes in biological condition along a gradient of increasing anthropogenic stress. As coral reefs collapse globally, scientists and managers are focused on how to sustain the crucial structure and functions, and the benefits that healthy coral reef ecosystems provide for many economies and societies. We developed a numeric (quantitative) BGC model for the coral reefs of Puerto Rico and the US Virgin Islands to transparently facilitate ecologically meaningful management decisions regarding these fragile resources. Here, reef conditions range from natural, undisturbed conditions to severely altered or degraded conditions. Numeric decision rules were developed by an expert panel for scleractinian corals and other benthic assemblages using multiple attributes to apply in shallow-water tropical fore reefs with depths <30 m. The numeric model employed decision rules based on metrics (e.g., % live coral cover, coral species richness, pollution-sensitive coral species, unproductive and sediment substrates, % cover by Orbicella spp.) used to assess coral reef condition. Model confirmation showed the numeric BCG model predicted the panel’s median site ratings for 84% of the sites used to calibrate the model and 89% of independent validation sites. The numeric BCG model is suitable for adaptive management applications and supports bioassessment and criteria development. It is a robust assessment tool that could be used to establish ecosystem condition that would aid resource managers in evaluating and communicating current or changing conditions, protect water and habitat quality in areas of high biological integrity, or develop restoration goals with stakeholders and other public beneficiaries.

## 1. Introduction

Healthy coral reef ecosystems provide a multitude of benefits (e.g., fishing, aquaculture, tourism, boating, education, coastal protection, culture, and bioprospecting) upon which many human economies and societies rely (Moberg and Folke 1999; Principe et al., 2012; Reguero et al., 2021; Storlazzi et al., 2019; van Beukering et al., 2011; Wilkinson, 2008). As coral reef condition has been declining globally over five decades (Hughes et al., 2007, 2018; Wilkinson 2008), the ecosystem goods and services they provide have been placed at great risk (Santavy et al., 2021; Woodhead et al., 2019). Sustaining coral reef ecosystems requires that crucial ecosystem functions are maintained (Harborne et al., 2017) and that reefs are protected from a variety of anthropogenic stressors (Darling et al., 2019; Williams et al., 2019). At local scales, exposure to sedimentation, nutrient enrichment, and turbidity degrade coral reefs (Hernández et al., 2009; Larson and Webb 2009; Loya 1976; Pollock et al., 2014); however, it has been difficult to assess causal relationships between terrestrial influence and coral reef degradation at regional scales (Fabricius 2005; Oliver et al., 2018; Smith et al., 2008). To improve protection of coral reef ecosystems, there is an urgent need for reliable tools to assess coral reef condition that reflect the cumulative impacts of multiple stressors; support adaptive management approaches; and facilitate communication among natural resource managers, stakeholders, and the public for transparent management decisions.

**Fig. 1.**
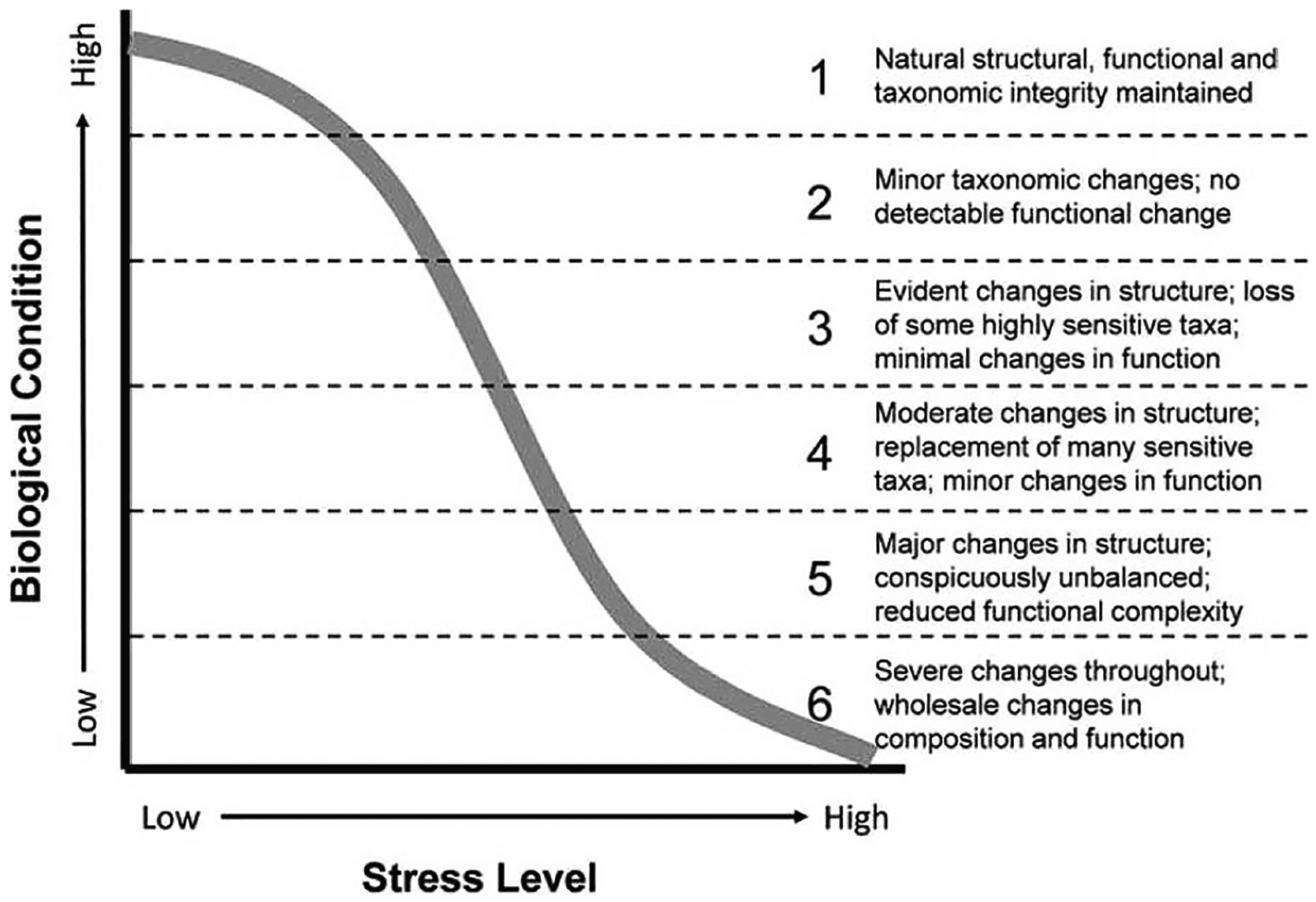
Conceptual model of the BCG relating biological condition on the y axis to level of exposure to stressors on the × axis (adapted from Davies and Jackson 2005).

The Biological Condition Gradient (BCG) is a robust assessment tool that can be used to assess biological condition and aid resource managers in evaluating current or changing conditions, protecting water and habitat quality in areas of high biological integrity, and developing restoration goals. The BCG is a conceptual model that describes incremental changes in biological condition along a gradient of increasing anthropogenic stress (e.g., physical, chemical, and biological impacts) (US EPA, 2016). Changes in biological condition are indicated by six levels that range from natural, undisturbed conditions (BCG level 1) to severely altered and degraded conditions (BCG level 6) (Fig. 1). BCG condition is depicted as departures from a natural or undisturbed state using observable biological and ecological attributes and metrics in the form of expert derived “decision rules” applied at each level. The BCG has been developed and applied to freshwater (Gerritsen et al., 2017; Hausmann et al., 2016; Paul et al., 2020) and estuarine (Shumchenia et al., 2015) systems to aid US jurisdictions (States, Tribes, and Territories) for compliance with the US Clean Water Act (US EPA, 2016). Santavy et al., (2016) proposed the BCG proof of concept for marine waters using reef-building corals and subsequently expanded the model into a qualitative, or narrative, BCG model for coral off the coast of Puerto Rico and US Virgin Islands (USVI; Santavy et al., in review). A companion BCG model for fish that inhabit coral reef ecosystems provides an additional tool to evaluate the quality of marine ecosystems (Bradley et al., 2020).

The narrative (qualitative) BCG for coral presented in Santavy et al. (in review) adapted the general BCG framework (US EPA, 2016) into narrative rules for Caribbean coral and established a basis for which a numeric (quantitative) BCG model could be developed. A qualitative BCG model is used to communicate reef condition relative to natural, undisturbed conditions. We present a numeric BCG model for Caribbean coral reefs, defined with assemblages of sessile benthic organisms, that can be used with the narrative coral BCG (Santavy et al., in review) and the reef fish BCG (Bradley et al., 2020) to comprehensively evaluate and communicate coral reef ecosystem condition. The narrative BCG model is used as a foundation and incorporates metrics obtained from various bioassessment surveys to create numeric rules for each BCG level. Our objectives were to develop numeric rules within the framework of the BCG to: assess reef condition; establish water-quality goals and restoration targets; measure incremental improvements in reef condition; and facilitate cross-agency communication. The numeric model provides a more robust process to inform management of the biological condition of coral reefs that can serve to ensure protection of high-quality marine waters and their biological communities, and to develop restoration targets. The model is suitable for adaptive management and supports bioassessment and criteria development. It provides a tool to develop biological criteria for coral reef ecosystems while formulating, justifying, and communicating management decisions with stakeholders and other public beneficiaries. An application of this model is demonstrated through a case study for St. Croix, US Virgin Islands.

## 2. Materials and methods

The quantitative BCG model was developed as a numeric expansion of the narrative BCG model using scleractinian corals and additional benthic reef-dwelling organisms. The process to develop, calibrate, and validate the narrative BCG model was described in detail in Santavy et al. (in review). Here, we focus on the methods used to develop the numeric rules for the quantitative model. Briefly, the steps are: 1) assemble and organize bioassessment data; 2) conduct data preparation and preliminary data analysis; 3) convene a panel of experts on coral reefs; 4) develop a numeric BCG model; and 5) test the model and review model performance. Using this framework, the expert panel rated the condition of coral reef sites, identified critical biological elements to develop numeric rules, and documented the rationale for their ratings. The ratings and rationale were translated into decision rules that were confirmed, adjusted, and iteratively recalibrated.

### 2.1. Step 1 assemble and organize bioassessment data

Data assembly required evaluation and examination of available bioassessment survey data into a high quality and spatially comprehensive dataset to identify sites that represented the full range of biological conditions. The datasets used to develop the narrative BCG rules described in Santavy et al. (in review) lacked quantitative measurements of benthic coverage that are critical to develop a robust numeric BCG model. Datasets were collated for the present study to include numeric metrics that could be used separately or in conjunction with the narrative rules. Bioassessment data with the requisite field measurements for metrics that could be calculated were available from NOAA’s National Coral Reef Monitoring Program (NCRMP) for Puerto Rico and USVI (Appendix A) (NOAA NCRMP 2013 USVI, 2013; NOAA NCRMP 2014 Puerto Rico, 2014). NOAA’s NCRMP surveys used underwater diver assessments on reefs in Puerto Rico and the USVI during 2013–2015 that applied a stratified random sampling design in shallow-water coral reefs (0–30 m). NOAA’s guidance documentation for NCRMP coral reef assessment in the US Caribbean territories is provided in detail in NOAA Coral Program (2014) and regularly updated with any modifications (NOAA 2015). The NCRMP bioassessment protocols, measurements, and metrics obtained for the present study are provided in Supplemental Information A and B. From these survey data, 57 fore-reef sites were used to calibrate the model and 18 different sites were used to validate it.

### 2.2. Step 2 analyze and prepare data

The NCRMP bioassessment data were prepared by computing metrics for each site, with primary emphasis on sessile assemblages (i.e., scleractinian coral communities). Predominantly two survey methods were used, a line point intercept (LPI) method that estimated the percent coverage of select benthic assemblages and a demographic survey protocol (DEMO) that estimated different metrics of coral community structure by assessing individual scleractinian colonies (Supplemental Information A). LPI benthic coverage was estimated using point counts along a 25 m linear transect for estimating ecologically important assemblages (e.g., macroalgae, turf algae, crustose coralline algae, corals, sponges) and substrate types (e.g., sand, rubble, hard bottom, bedrock) (benthic categories in Table 1).

Briefly, the DEMO survey method measured individual coral colonies to obtain maximum colony diameter, maximum height, coral species, and percent tissue mortality using a linear quadrant census and binned by coral species. Most coral metrics were based on assessment of each individual colony surface of skeletal area (CSA) only using individual colonies>4 cm diameter, excluding the basal areas attached to the substrate. Live colony surface area (LCSA) was the amount of tissue covering the CSA or skeletal surface area (cm^2^) (Fisher et al., 2019; Santavy et al., 2012). CSA calculations were adjusted for coral species using a specific colony morphology factor (Supplemental Information B, Table B1) to estimate the 3-dimensional exterior colony surface area. For each survey site, metrics included average CSA/colony and LCSA/colony for each species and for all species combined. CSA and LCSA are not directly comparable to the planar % coral cover standardized by species reported in many past studies (Hill and Wilkinson 2004; Jokiel et al., 2015). A comparable metric to % coral cover was calculated from these survey data to provide an estimate of 2-dimensional planar (2D) live coral surface area (LCSA_2D). LCSA_2D used maximum colony diameters to calculate the planar area of each colony and summed for a total 2D percent coral cover by species (Supplemental Information B). Additional metrics calculated from DEMO data included: mean colony densities by species; colony density of organisms exhibiting disease or bleaching; mean percent old or new coral mortality by species (Lang 2003; Santavy et al., 2012); mean, maximum and minimum colony diameter (cm) by species; and maximum colony height (cm) by species. Health metrics for scleractinian coral were prevalence of disease, partial bleaching, and total bleaching.

A common subset of metrics was calculated from both the LPI data and the DEMO data to test whether each survey-specific metric helped to discriminate different BCG levels. The common metrics were: coral species richness; % coral cover by species; proportional percent of each species from the total species expressed as either % (LPI) or abundance (DEMO); and the proportional percent of taxa that were tolerant or sensitive to a particular stressor. The abundance of ecologically and commercially important species were provided for: Caribbean spiny lobsters (*Panulirus argus* and *Panulirus guttatus*); queen conch (*Aligers gigas*); long-spined sea urchins (*Diadema antillarum*); and presence/absence of seven threatened and endangered scleractinian species. Abundances of threatened coral species, including *Acropora cervicornis* and *Acropora palmata*, were recorded in the 2013 NCRMP surveys (NOAA 2015). In 2014, five more Caribbean coral species were determined to be threatened and were added to the survey data collected: *Dendrogyra cylindrus, Orbicella annularis, Orbicella faveolata, Orbicella franksi,* and *Mycetophyllia ferox* (NOAA 2014).

Responding to the irregularities for coarse rugosity measurements using the chain-length method (Rogers et al., 1994), NCRMP developed a measure for rugosity or topographical surface heterogeneity at a finer scale (Brandt et al., 2009). No underwater videos were available for the NCRMP surveys and limited underwater photographs were available for most stations.

### 2.3. Step 3 convene an expert panel:

Experts were selected to serve on a panel to calibrate and validate a BCG model using benthic assemblages from coral reefs. The panel members were intentionally chosen to collectively represent a breadth of expertise in coral-reef bioassessments, marine ecology, coral reef biology, and taxonomy; and they were all experienced in Caribbean and western Atlantic reefs. Experts were chosen to represent a diverse membership affiliated with state, territorial, federal, academic, and non-governmental organizations to minimize internal bias (US EPA, 2016). The panel members had a range of experience in coral reef ecosystems from five to over 40 years. The collective set of experts that comprised the panel are herein referred as “the panel”; when experts performed activities individually, they are referred herein as an “expert” or “the experts” (Supplemental Information C). The panel’s objective was to develop biological assessment endpoints from the metrics provided by the survey data that described coral reef condition across BCG levels 1–6. The reef habitat classification system in Costa et al., (2009), Costa et al. (2013) was used, which is based on reef types, geographic zones, and geomorphological structures to identify sites in the fore reef-slope zone (the area along the seaward edge of reef crest of a barrier or fringing reef that slopes into deeper water). Only sites in the fore-reef slope zone dominated by the reef-building coral genus *Orbicella* (Williams et al., 2015) were used in the development of this model.

Ten BCG attributes are defined in the BCG framework for all environments and include taxa sensitivity, organism condition, and various ecosystem functions that are responsive to taxa structure and compositional changes when exposed to major anthropogenic stressors (Davies and Jackson 2006; US EPA, 2016) (Supplemental Information Table D1). A total of 46 Caribbean coral species and three hydrozoan species with calcareous skeletons were assigned to one of the BCG attributes I-VI (herein represented by Roman numerals) based on their sensitivity or tolerance to pollution (I–V) or whether the species was non-native (VI) (Santavy et al., in review). If the data did not support an attribute assignment, the taxa were not assigned to an attribute category. BCG attributes VII–X were not used in this study as the information needed to inform them is not fully developed for coral reef assemblages. Species sensitivity and tolerance toward anthropogenic stressors were based on elevated sea temperature and exposure to sediments (Supplemental Information D, Table D2). The latter was used as a proxy for land-based sources of pollution. Since data on the tolerance of coral species to different anthropogenic stressors are limited, these assignments were based primarily on expert knowledge and panel consensus. No assignment was made for species that the majority of the experts on the panel did not agree in its assignment. The number of species assigned to each BCG attribute were: Attribute I (Historically documented, sensitive, long-lived, or regionally endemic taxa): 0 species; Attribute II (Highly sensitive taxa): 2 species; Attribute III (Intermediate sensitive taxa): 9 species; Attribute IV (Intermediate tolerant taxa): 19 species; Attribute V (tolerant taxa): 13 species; Attribute VI (non-native taxa): 1 species. There were 5 species not assigned to an attribute based on insufficient knowledge by experts and in literature.

### 2.4. Step 4: Develop BCG decision model for numeric rules

The premise of the quantitative benthic BCG model is based on the structural and functional importance of benthic organisms (including reef-building corals, algae, and other sessile invertebrates), how they interact, and how they indicate overall reef condition. The development or calibration of the numeric model produced a multiple attribute decision model that simulates the consensus expert decisions based on a set of quantitative rules using the calibration data. The model was developed as a set of decision rules for each BCG level that use numeric thresholds for the metrics defined in Step 2. The decision rules for a single BCG level were not typically based on a single metric (e.g., % coral cover) but included other metrics (reef building species, species richness, etc.), such that each BCG level was defined by multiple rules. To calibrate the model, the expert panel was provided the site data, the metrics calculated in Step 2, and limited photos if available for that site. First, they ascertained whether the site was a fore-reef slope zone dominated by *Orbicella* species complex, verified with aerial imagery using Google Maps (accessed 2012 to 2016) to ensure the reef was in the correct position in relation to the entire reef complex geomorphology; within a plausible depth range; and seaward of the reef crest.

Fore-reef sites were assigned a BCG condition level by each expert ranging from level 1 - natural, undisturbed by anthropogenic stressors to level 6 - severely altered from natural. Each expert individually rated the biological condition of each site by considering the BCG level generic descriptions, site data metrics provided, and narrative decision rules for the benthic model presented in Santavy et al. (in review). Each expert documented their rationale, logic, and the factors with the greatest weight for basing their decision and assigning an integer BCG level score of 1–6. The panel also requested to include intermediate levels as ‘ + ‘ or ‘ − ‘ to indicate when a site exhibits some characteristics of the next best or worse condition but not great enough to assign the site to the better or worse level. Scores were counted as the BCG integer face value and either added (+0.33) or subtracted (−0.33) to numerically translate the ‘−’ and ‘+’, respectively. For example, a site could be rated level 3+ (score = 2.67) by a single expert if it was a very good level 3 but not meeting the expert’s expectation for a level 2. Conversely, a site could be rated level 3− (score = 3.33) if it was a poor level 3 but better than level 4. The ‘+’ and ‘−’ scores assisted the expert panel in articulating the thresholds between different BCG levels.

The panel considered the community structure and function among different assemblages to make a BCG level assignment using the metric values provided for the site. Each expert’s ratings and associated logic/rationale were compiled as individual scores to share during facilitated discussions, when each expert was provided the opportunity to maintain or change their initial BCG level score as part of the iterative process. As the panel evaluated more sites and assigned BCG condition levels, change-points and boundaries of uncertainty emerged as patterns of metrics translated into ecologically meaningful decision rules for each BCG condition level. For each site, a panel median BCG score was calculated from all the experts’ individual ratings using the scores described in preceding paragraph.

To ensure the numeric decision rules yielded consistent assignments of sites across BCG levels, it was necessary to formalize and quantify the expert knowledge by codifying the BCG level descriptions into a set of quantitative rules (e.g., Droesen 1996; Gerritsen et al., 2017). The logic used to make the experts’ decision was described by expressing the critical ecological traits derived from the site metrics provided to each expert such as: taxa richness and density; percent coral cover; presence of taxa that were tolerant or sensitive to a particular stressor; type and percent of coral mortality; amount of algal cover and bare substrate; and other measurable observations described in Step 2. Numeric decision rules were expressed as statements that related metric nominal values to BCG level descriptions by converting the BCG level ratings to combinations of numeric rules. Decision rules are logical statements that experts use to make decisions; they need to be clear so that any person with knowledge of coral reefs can follow them to obtain the same BCG level score as the experts. These practices allowed the decision criteria to be transparent and ecologically meaningful (US EPA, 2016). The BCG framework process was iteratively applied, reviewed, and revised until the panel was confident that the model replicated their decision processes and accurately predicted the same BCG condition level that they assigned as the panel median score.

Mathematical fuzzy logic (Zadeh, 2008; Supplemental Information E) was used to develop an inference model to replicate the experts’ decision process (US EPA, 2016). The decision rules for the numeric model defined quantitative thresholds based on nominal metric values provided and tested for their discriminatory power to detect differences between BCG levels at these sites. Boxplots were developed for each metric to determine rule thresholds and range values that discerned differences between BCG levels. For each metric and BCG level, the boxplots showed the median, interquartile range, non-outlier ranges, outliers, and extreme values. Mathematical fuzzy set theory was applied to interpret the box and whisker plots by comparing calculated metric median and percentiles to the panel’s median BCG score for site assignments (Zadeh, 1965, Zadeh, 2008). Mathematical fuzzy set theory can be used to interpret “irreducible measurement uncertainty” by capturing the vagueness of terms such as ‘many’, ‘large,’ or ‘few’; and enhancing the ability to model human reasoning and decision making (Zadeh, 2008; Supplemental Information E, Figure E1). Analyses were computed in Statistica version 7.1 (TIBCO Software Inc., Palo Alto, CA, USA).

Each metric was defined quantitatively by plotting the metric values from all the sites binned by each BCG level as a fuzzy set. Every metric-based rule within each BCG level was tested individually to determine whether the measured value had an inclusion membership function (MF) that determined whether it was fully contained in the set (1.00) or completely excluded (0.00) (Gerritsen et al., 2017). For a given rule, the membership value (MemV) of a metric value (MV) was the linear interpolation between the maximum membership value (MaxV) and minimum membership value (MinV) of the MF and normalized to zero to derive the value for a metric MemV. If the BCG rule had a MF that stated the variable value must be > or ≥ a certain number, the MF of that variable followed these rules: if MV ≥ MaxV then MemV = 1.00; if MV ≤ MinV then MemV = 0.0; and if the MV = nominal MV (rule value at the midpoint between MinV and MaxV), the MemV = 0.5. Alternatively, if the rule had MF that the variable value must be < or ≤ a certain number. In such cases, the MF followed the rules: if MV ≤ or < MinV then MemV = 1.00; if MV ≥ MaxV then MemV = 0.00; and if MV = nominal MV (rule value also the midpoint between MinV and MaxV), then MemV = 0.5 (see Supplemental Information E).

When the metric patterns from the boxplots matched the panel’s narrative model statements developed for each BCG condition level, that metric and corresponding values were considered a good candidate to include in the numeric model to distinguish it from the previous or next BCG level. If the metric patterns did not match the narrative model statements provided to them, the panel deliberated to identify why the numeric data did not support the narrative model rule. They examined whether that metric responded to natural factors that had not been considered, if the metrics might not have been calculated as experts intended, or if there were confounding or compounding factors the experts did not initially discern.

Decision rules were assigned BCG condition levels 1–6 and were applied as a logical cascade for any given site (More details in Supplemental Information E, Figure E2). Since there were no BCG levels 1 and 2 sites represented in the surveys, site characteristics were initially compared to the decision rules in BCG level 3 to determine if the required rules were met. If the site failed to meet all the required rules at BCG level 3, the site characteristics were compared to BCG level 4 decision rules. If the rules failed to match BCG level 4 then the site characteristics were compared to BCG level 5 decision rules. If these decision rules could not be met, the site was assigned to BCG level 6.

A set of guidelines used MemV for each set of BCG level decision rules to assign a BCG level to each site. If the partial MemVs for two BCG levels were within 0.10 of each other, then the prediction was a tie between the two levels. For example, when the MV for BCG level 2 = 0.55 and for BCG level 3 = 0.45 this indicated a BCG level 2–3 tie. If the MV of a site for BCG level 3 = 0.70 and level 4 = 0.30, the site was scored BCG level 3 −. Alternatively, if the BCG level 3 = 0.2 and level 4 = 0.8 the site was scored BCG level 4 +. When the MemV > 0.90 for any BCG level, it indicated that the rules met that BCG level without any qualifying ‘+’ or ‘ − ‘. After formulating the rules, rule thresholds, and combination rules, the model was presented to the panel for approval or adjustment.

### 2.5. Step 5: Test model and review model performance

To test the performance of the calibrated model a confirmatory or validation process used different data for 18 independent sites not used in model calibration. These sites were evaluated separately by the experts to determine score consistency among the experts and model performance. Model performance was evaluated by comparing the model-predicted membership values for the metrics defined in each BCG level for each quantitative decision rule with the initial BCG level’s panel median value assigned to that site. The number of sites that matched the BCG decision model’s nominal level exactly with the panel’s BCG median value (matched sites) was compared to the number of sites when the model predicted a BCG level that differed from the panel’s BCG median value (mismatch sites). For the mismatched sites, the differences between the model and panel assignments used a weighted concordance measure between the quantitative model prediction and the panel ratings assigned. If the model predicted a BCG level tie that did not match the panel’s assigned value, or vice-versa, a difference of half BCG level was assigned to each of the preceding and proceeding levels. These values reflected the accuracy of the model as applied to multiple validation sites and determined if there was a directional bias between the predicted BCG model value and the panel value (i.e., Did the BCG model consistently rate sites better or worse than the panel?). This same scoring system was applied to determine the precision of the individual experts rating compared to the median panel BCG scores for each validation site as described in Step 4 for model calibration.

## 3. Results

The coral reef BCG model for benthic macroinvertebrate assemblages includes expert-derived narrative descriptions for each BCG level, 1 through 6 (Santavy et al in review). There were no sites used in numeric model development that the experts judged as a BCG level 1 or 2. As a result, the numeric model discriminates between levels 3, 4, 5 and 6. The narrative BCG level 1 and 2 descriptions provide a template for further testing and development of numeric decision rules should undisturbed or minimally disturbed sites be found.

### 3.1. Assembled data

The panel reviewed 66 sites from the NCRMP data set, but only used 57 sites for BCG model calibration. Sites were excluded if they did not contain both LPI and DEMO survey data (Table 2). The sites were from Puerto Rico and the US Virgin Island from deep (>12 m) and shallow (<12 m) water habitats.

### 3.2. Calibration of BCG decision model for numeric rules

**Fig. 2.**
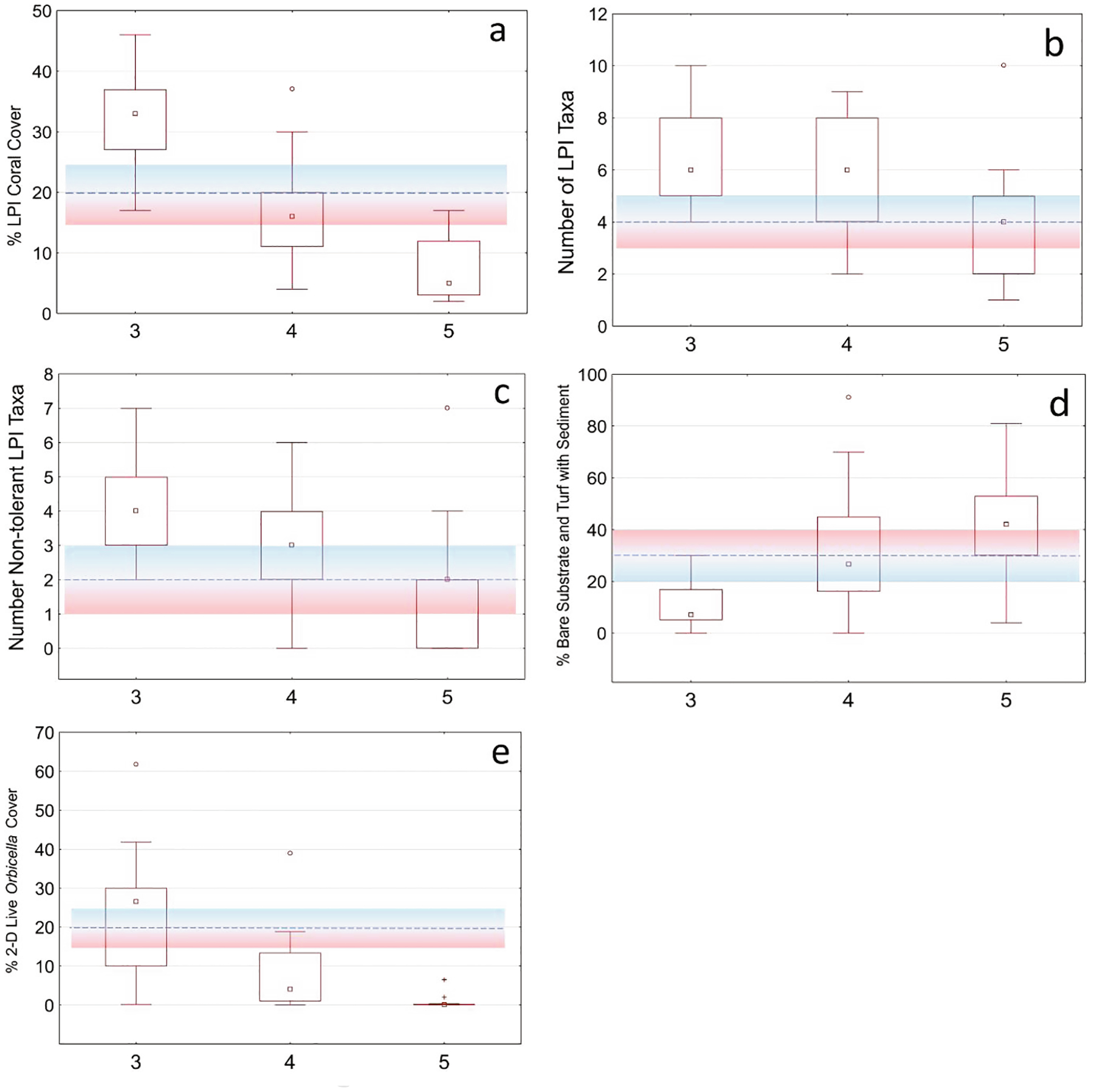
Summary of metric values in numeric rules used for discriminating between benthic BCG levels 3 and 4. Metrics shown in each graph: a) % coral cover (LPI), b) # coral spp. (LPI), c) # non-tolerant coral spp. (LPI), d) % unproductive cover (LPI), and e) % live *Orbicella* (DEMO). The dashed line showed the rule thresholds and ranges shown in the color-shaded region. Membership values were calculated as 1.0 if the metric value is better than the blue range, 0.0 if worse than the red region, and partial membership between 0.0 and 1.0 if within the shaded region. Distributions included the median (central square), interquartile range (rectangular box), non-outlier ranges (whiskers), and outliers (circular marks).

The numeric decision rules showed a pattern of decreasing percent coral cover and lower percent live tissue on individual coral colonies with increasing BCG level. As the biological conditions of the reef deteriorated, there were increases in mortality of coral tissue and colonies, and an increased presence of bare substrate and turf algae with sediment accumulation. Similarly, as the condition of reefs declined, the number of decision rules that described condition mostly decreased until BCG level 6 was defined by virtual absence of most taxa found in BCG levels 3–5. When the decision rules and logic of the panel were compared to metric statistical summaries displayed as boxplots, the most consistent and discerning metric was % coral cover (LPI) that detected differences between BCG levels 3 (median value = 33%), 4 (16%), and 5 (7%) (Fig. 2a).

Eleven metrics significantly contributed to condition changes and were included in the benthic numeric model (Summary statistics in Supplemental Information F, Table F1). Seven metrics monotonically decreased as reef condition declined (% coral cover (LPI); # non-tolerant coral spp. (LPI); % live *Orbicella* (DEMO); % non-tolerant coral cover (LPI); density med-large colonies (DEMO); live coral cover 3D (DEMO); and % *Orbicella* cover (LPI)). The unproductive benthic cover was comprised of bare substrate and turf algae with sediment cover. This cover type increased as reef condition declined. Metrics used in the narrative model that successfully transferred to the numeric model were: % coral cover (LPI) also described in the narrative model as % coral cover (planar); colony density (DEMO); # coral spp. (LPI) or species richness; and live coral cover 3D (DEMO) or colony tissue-surface area (Santavy et al., in review).

BCG level 3 sites contained moderate coral cover of species sensitive to moderately tolerant to sediment stress, and low benthic coverage with bare substrate or sedimented algal turf considered unproductive. Five decision rules discerned between BCG condition levels 3 and 4 (Fig. 2). Most metric rules were derived from LPI survey data (e.g., % coral cover, # non-tolerant coral spp., # coral spp, and % unproductive cover), with fewer rules derived from the DEMO survey data (e.g., % live *Orbicella*). The nominal value for the % coral cover was > 20% (15–25). The MemV = 0.5 for 20%, with increasing MemV to a maximum of 1 for % coral cover (LPI) ≥ 25% or decreasing MemV to a minimum of 0 for % coral cover ≤ 15% (Table 3). Four additional metrics discerned membership in BCG level 3: # coral spp. > 4 with minimum threshold values of 3 species and ≥ 5 species (Fig. 2b), # non-tolerant coral spp. with decision rule > 2 (3–5) (Fig. 2c), and % unproductive cover < 30% (20–40) (Fig. 2d). Metrics membership values of all the first four rules must be true to be scored as BCG level 3. One exception could override compliance with these first four rules, only if the fifth rule was true that % live *Orbicella* > 20% (15–25) (Fig. 2e). The panel determined rule 5 to be a dependable indicator of relatively undisturbed reef conditions. If none of these rules for BCG level 3 were met, the numeric rules for BCG level 4 were evaluated.

**Fig. 3.**
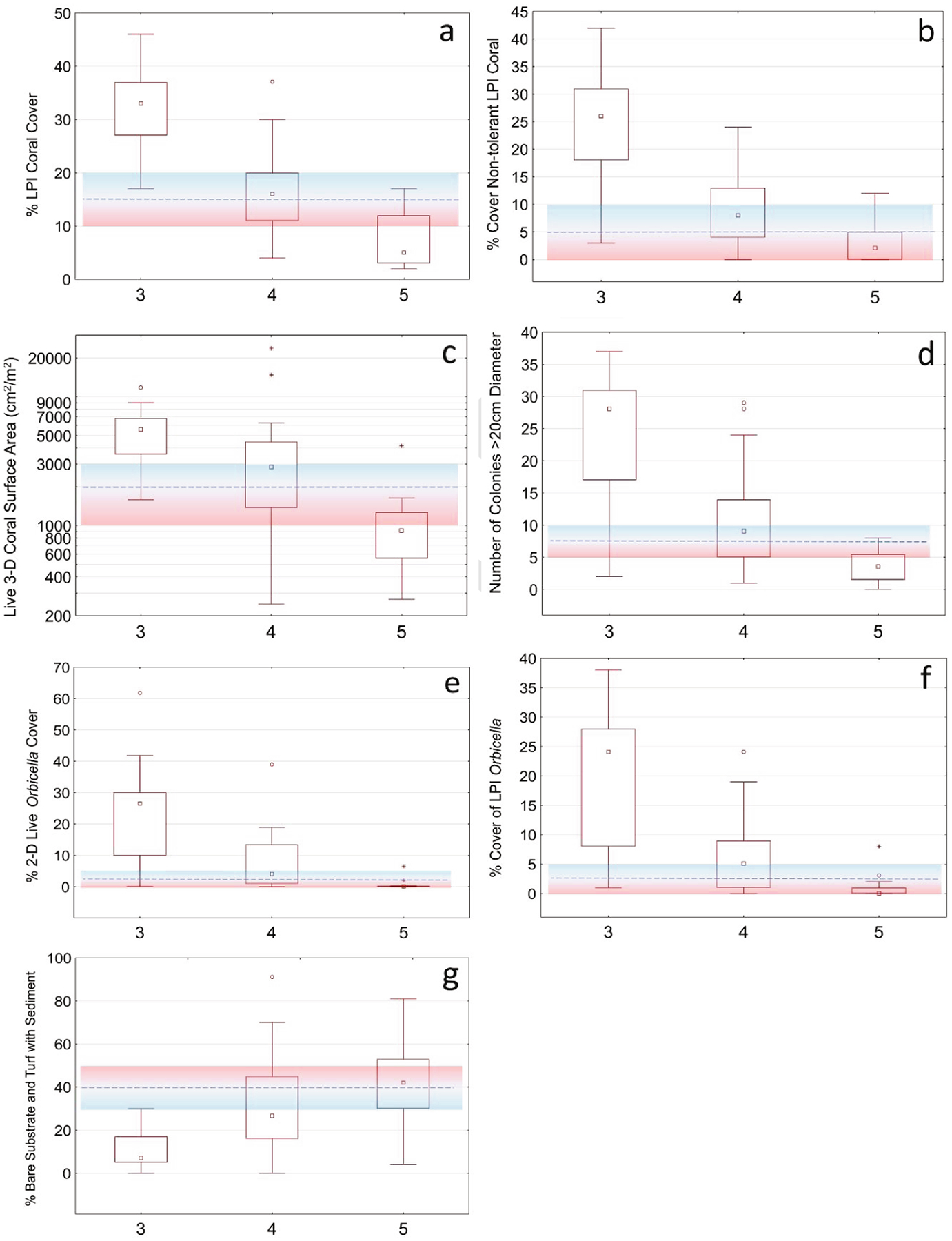
Summary of metric values in numeric rules used for discriminating between benthic BCG levels 4 and 5. Metrics shown in each graph: a) % coral cover (LPI), b) % non-tolerant coral cover (LPI), c) live coral cover 3D (DEMO), d) density med-large colonies (DEMO), e) % live *Orbicella* (DEMO), f) % *Orbicella* cover (LPI), and g) % unproductive cover (LPI). The dashed line showed the rule thresholds and ranges shown in the color-shaded region. Membership values were calculated as 1.0 if the metric value was better than the blue range, 0.0 if worse than the red region, and partial membership between 0.0 and 1.0 if within the shaded region. Distributions included the median (central square), interquartile range (rectangular box), non-outlier ranges (whiskers), outliers (circular marks) and extremes (stars).

BCG level 4 represented biological condition with significant alteration in assemblage composition including loss of sensitive taxa and declining ecosystem function. Low to moderate coral cover was expected and measured by declining values in both percent cover and live tissue cover, highlighting the importance of the reef building coral genus *Orbicella* (Fig. 3). Seven numeric rules discriminated BCG level 4 from 5, but only three rules must be true to be assigned to BCG level 4. Three metrics that defined BCG level 3 rules were also discriminatory for BCG level 4, % coral cover (LPI) (Fig. 3a), % live *Orbicella* (DEMO) (Fig. 3e), and % unproductive substrate (LPI) (Fig. 3g). Other metrics used in decision rules reflected colony maximum diameter sizes (density med-large colonies (DEMO)) (Fig. 3c) and the percent cover of non-tolerant LPI coral species (% non-tolerant coral cover (LPI)) (Fig. 3b). The % coral cover (LPI) numeric rule for low to moderate coral cover was defined as MV > 15% (10–20), although at least > 2.5% (0–5) must be of the genus *Orbicella* (Table 3). If fewer than three rules were true, then the numeric rules for BCG level 5 were considered.

**Fig. 4.**
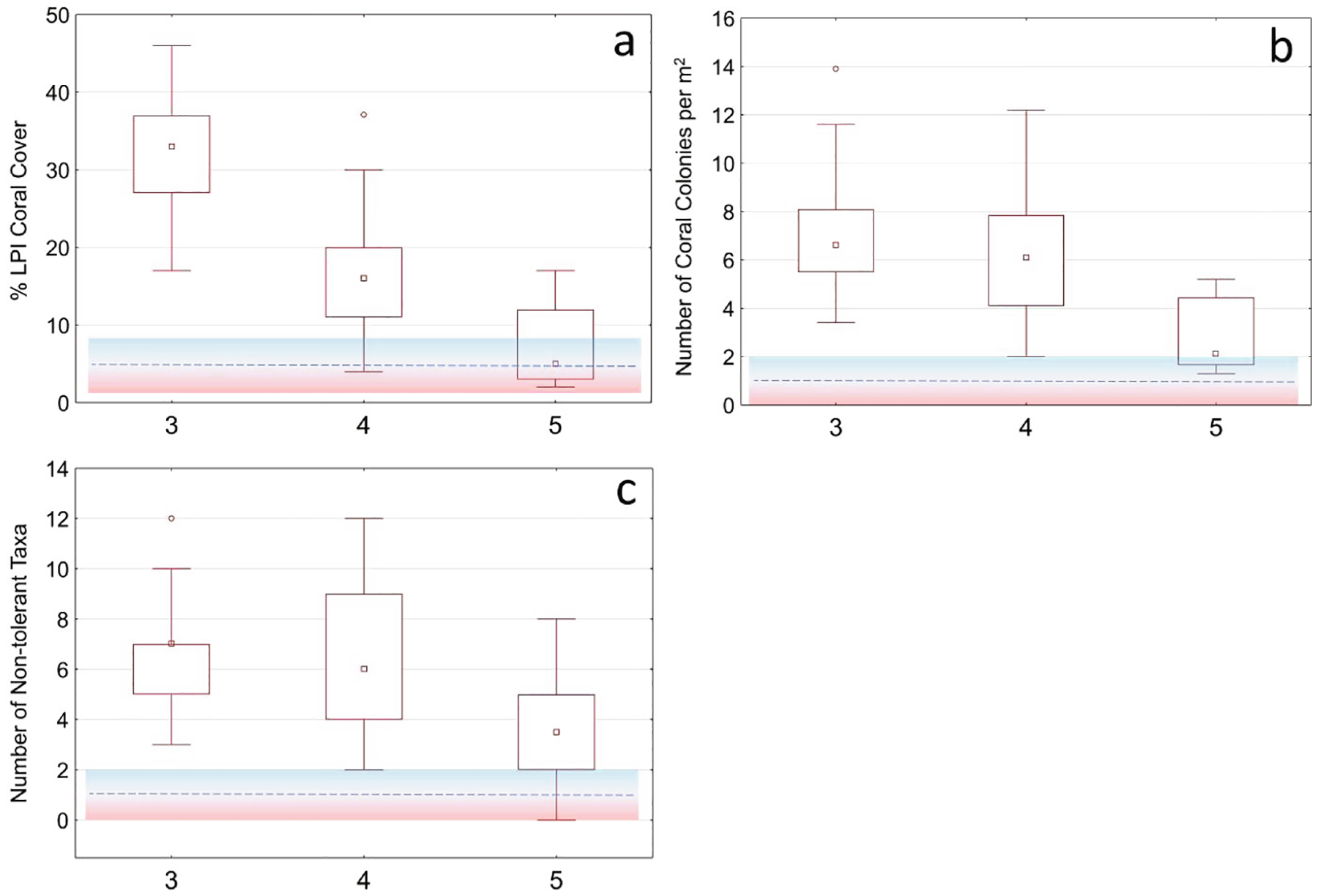
Summary of metric values in numeric rules used for discriminating between benthic BCG levels 5 and 6. Metrics shown in each graph: a) % coral cover (LPI), b) colony density (DEMO) and c) # non-tolerant coral spp. (DEMO). The dashed line showed the rule thresholds and ranges shown in the color-shaded region. Membership values were calculated as 1.0 if the metric value was better than the blue range, 0.0 if worse than the red region, and partial membership between 0.0 and 1.0 if within the shaded region. Distributions included the median (central square), interquartile range (rectangular box), non-outlier ranges (whiskers), and outliers (circular marks).

BCG level 5 had significantly reduced structural and functional complexity defined by three decision rules of which two must be true for inclusion of BCG level 5. For sites to be assigned to BCG level 5 rather than level 6, there must be some live coral cover, and it be comprised of sensitive or moderately tolerant coral species (Table 3). BCG level 5 was characterized by minimal coral cover (% coral cover (LPI)) (>5% (2–8)) (Fig. 4a), and the lowest density of coral colonies (colony density (DEMO)) (>1 colony/m^2^ (0–2)) (Fig. 4b) of which at least one species of non-tolerant coral taxa must be present (# non-tolerant coral spp. (DEMO)) (>1 species (0–2)) (Fig. 4c). If a site did not meet BCG level 5 rules, then it was assigned to level 6.

### 3.3. Test model and review model performance

**Fig. 5.**
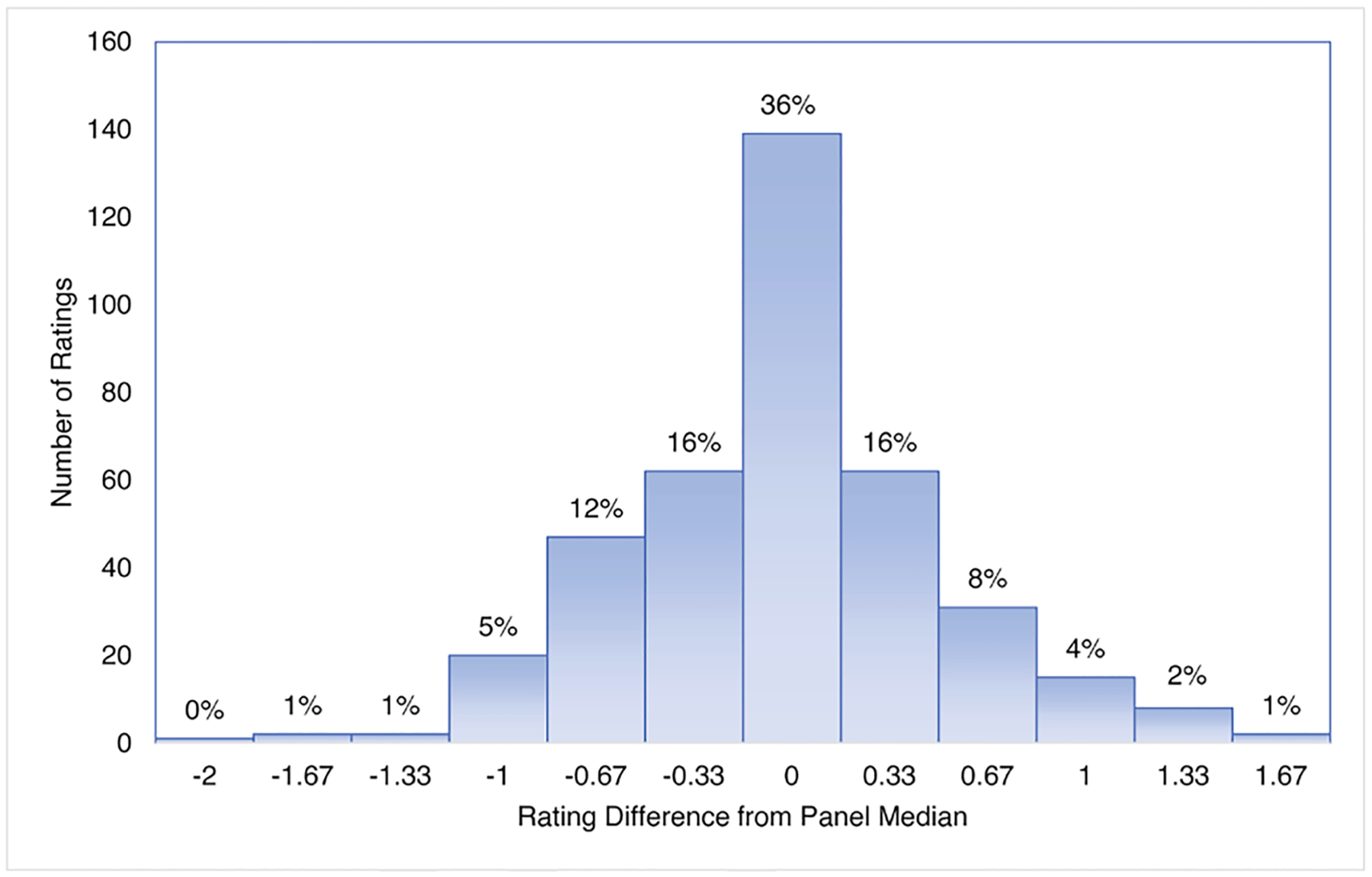
Precision of individual ratings for the BCG model calibration samples, measured as the difference between the sample’s median BCG level and the expert’s individual rating. Increments of ± 0.33 represent differences that included “+”, and “−” ratings.

**Fig. 6.**
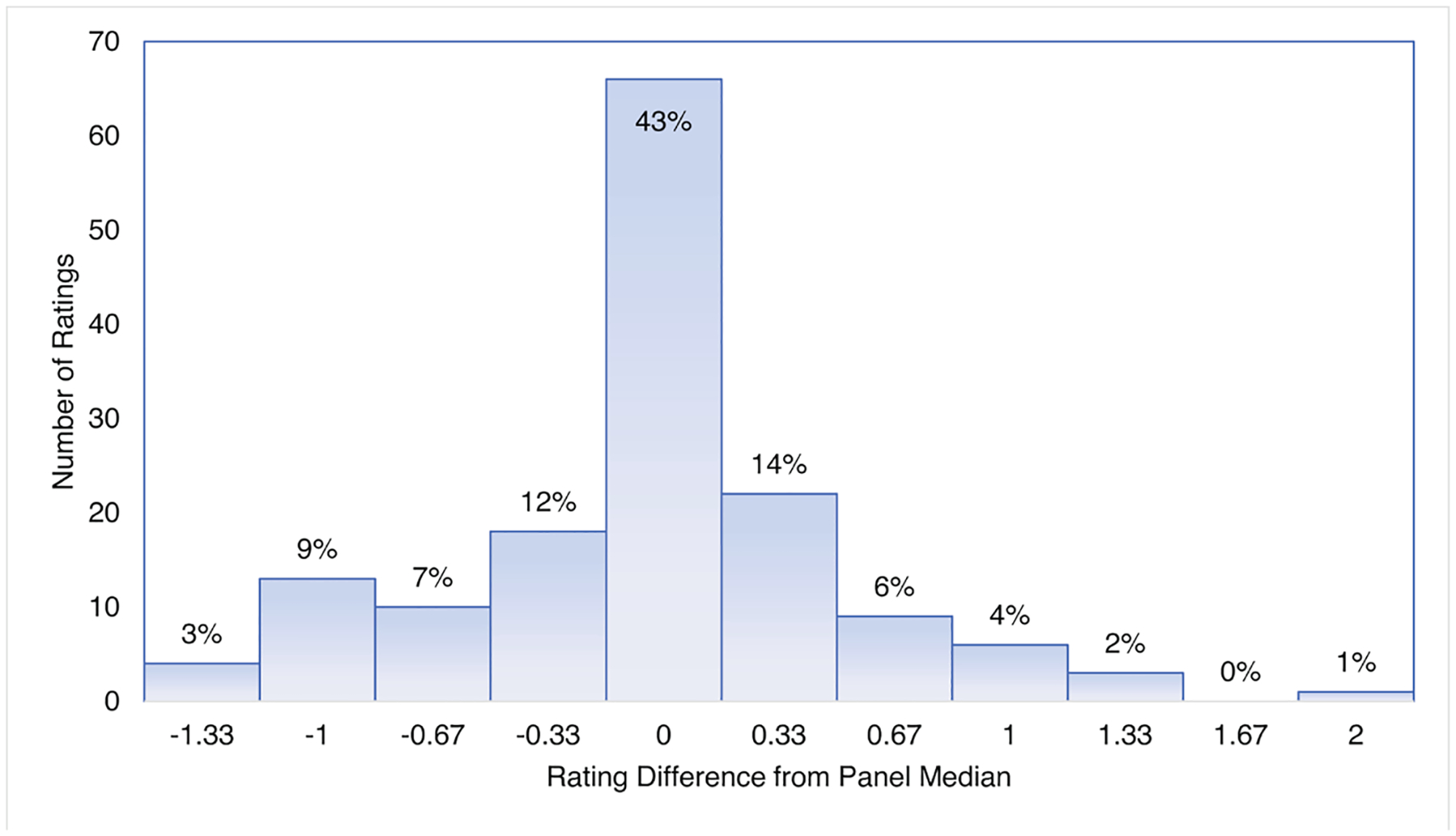
Precision of individual ratings for the BCG model validation samples, measured as the difference between the sample’s median BCG level and the expert’s individual rating. Increments of ± 0.33 represent differences that included “+” and “−” ratings.

There was high precision of the individual expert’s ratings centered around the panel median value for both the 57 calibration and 18 validation sites. Precision for the calibration sites used 392 individual ratings and showed 68% of the expert ratings were within a one third of a BCG level from the panel median value which, for comparison, was the difference between the integer BCG level and either a ‘+’, and ‘−’ (Fig. 5). Most individual ratings (96%) were within one BCG level of the panel median values. Only one rating was two BCG levels different than the site median. Similarly, there was high precision for 18 validation sites that used 152 individual ratings to show 63% were within a third of the panel median value (Fig. 6). Most individual ratings (95%) were within one BCG level of the panel median value. The greatest difference between the panel median value and the expert-assigned BCG levels was for one rating in which there was a two-BCG level difference.

**Fig. 7.**
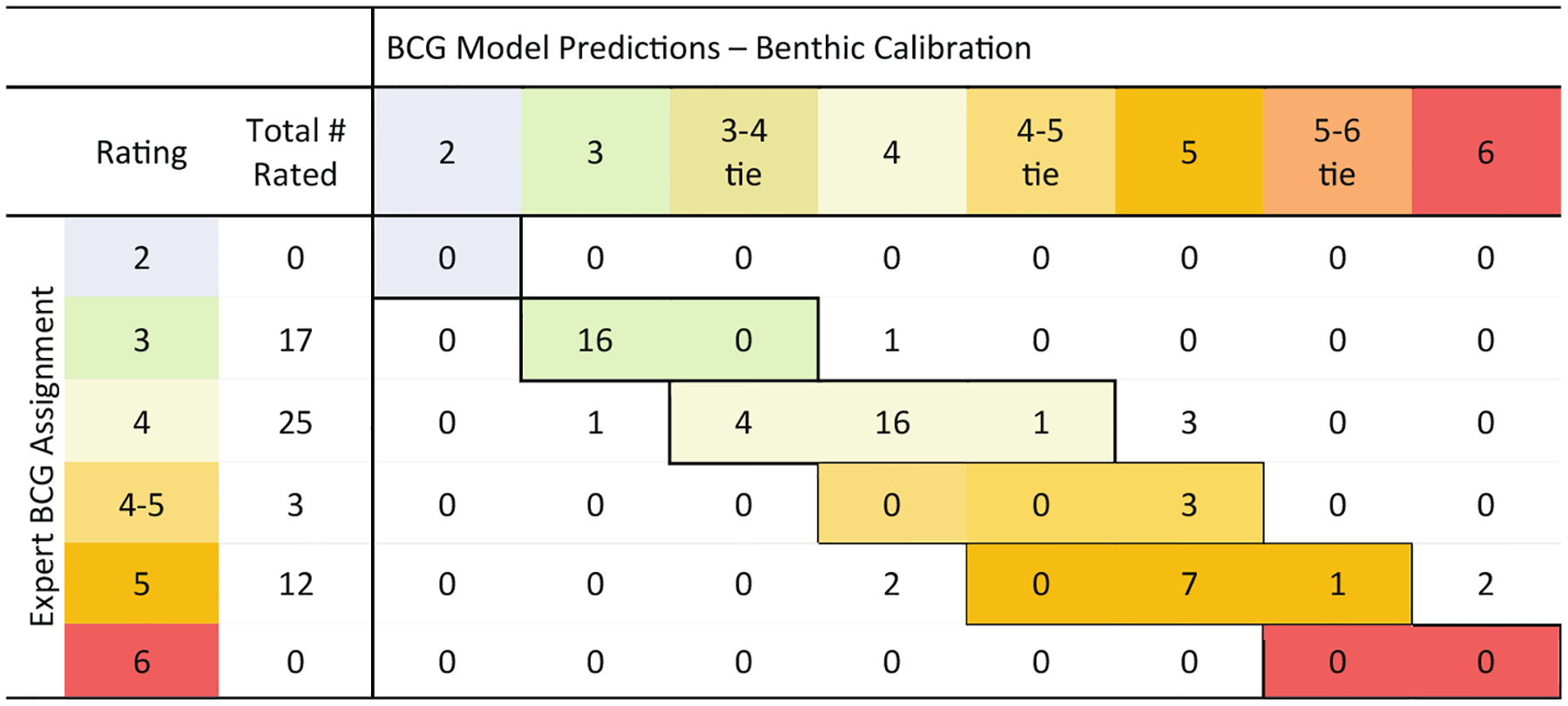
Comparison of expert assignments to BCG levels for benthic calibration of reef samples compared to BCG levels predicted by the model. Cells showed where there was agreement (shaded cells) and differences (unshaded cells).

Comparison of the panel median BCG level assignments to those predicted by the numeric model for the calibration reef sites showed high agreement (shaded or matched cells) and low disparity (unshaded or mismatched cells) (Fig. 7). Out of 57 calibration sites, the numeric model predicted the same BCG level as the panel median value for 48 sites (84%). There were nine predictions counted as correct that were tied between levels either in expert assignment or model prediction (16%). The prediction model was counted as an error at four sites, although the difference from the assignment was very similar (7%). For example, a site that was assigned BCG level 3 − by the expert panel may be very similar to a predicted level 4+, but because they are in different BCG levels, the model prediction was counted as an error. No model prediction was more than one BCG level different from the panel’s median value assignment.

**Fig. 8.**
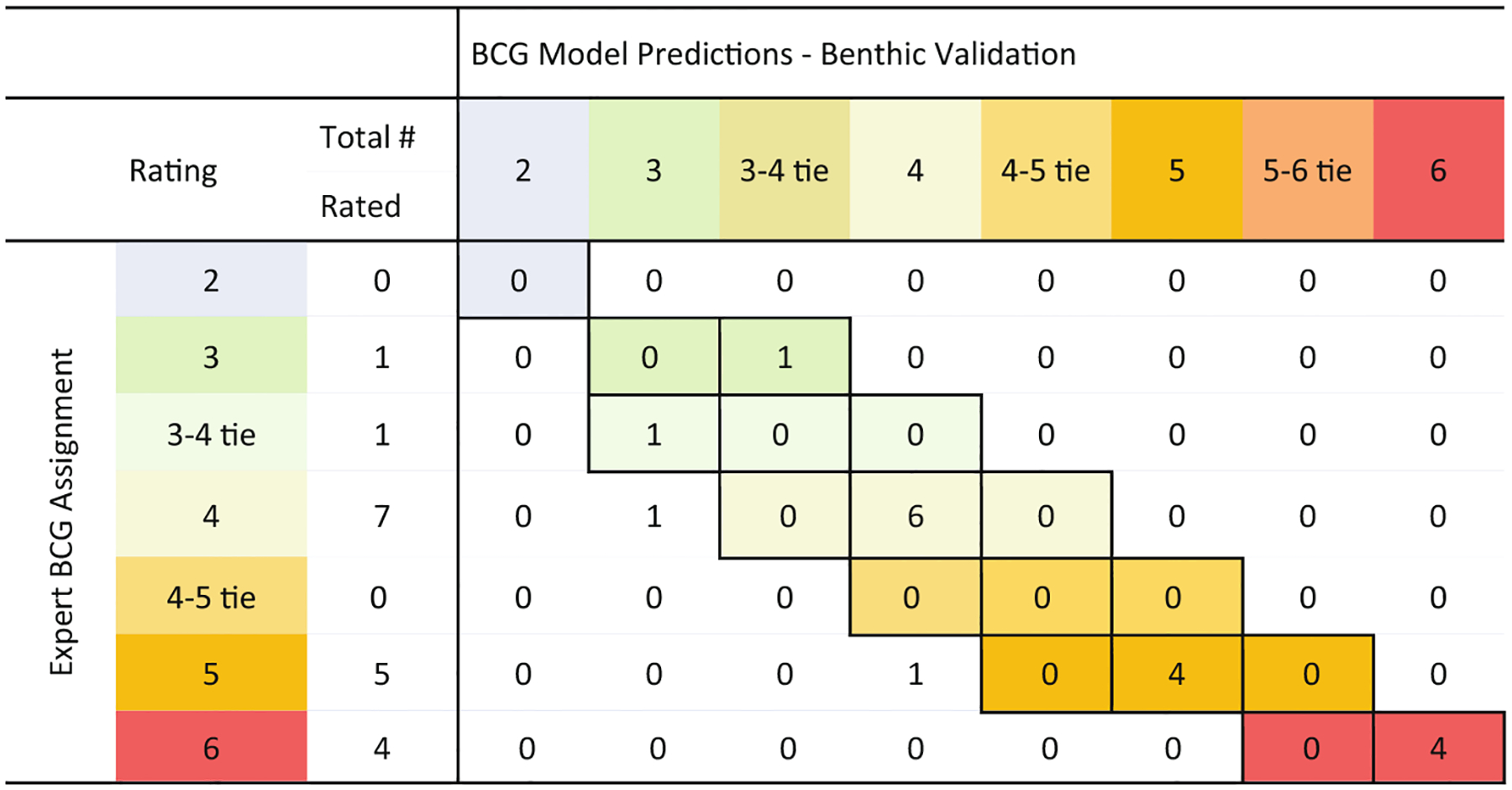
Comparison of expert ratings to BCG levels for benthic validation reef samples compared to BCG levels predicted by the model. Cells showed where there was agreement (shaded cells) and differences (unshaded cells).

Comparison of the panel median values for BCG level assignments to those predicted by the numeric model for the validation reef sites also showed high agreement and low disparity. All but two of the 18 sites matched the panel median value for 89% agreement (Fig. 8). For one site, the site median BCG value was level 5 while the model predicted a level 4 condition. The other site was rated as a level 4 by the panel, but the model predicted a level 3 because there was>25% coverage of live *Orbicella* colonies (% live *Orbicella* (DEMO)). Though other rules at level 3 failed, this rule was applied using “or” logic that over-ruled the requirement to have agreement of four rules

## 4. Case study example for model application

This case study demonstrates how to apply the BCG numeric model to predict the condition of coral reefs using benthic assemblage data. The site was an aggregate coral reef found on the fore-reef side in St. Croix, USVI from NOAA NCRMP dataset used to calibrate the numeric model. The site represented a shallow depth stratum ranging from 9.4 to −10.7 m. Data for the site were analyzed as described in Step 2. Site metric values and characteristics included BCG attribute-based, coral cover, and other benthic assemblage metrics (Supplemental Information G, Figures G1–G4). Here, we demonstrate how to calculate and interpret membership values (MemV) to accept or reject individual decision rules first, and then how to apply the suite of MemVs for decision rules to assign a BCG condition level to this site. MemVs were calculated using the appropriate formula dependent upon whether the full membership value of 1 was greater than the metric nominal value or less than the metric nominal value (Supplemental Information E). The first five decision rules that define BCG level 3 have MVs and their corresponding MemV calculations for this site shown in Table 4.

The first five rules were tested to determine which metrics had MemV > 0 to decide whether to assign the site to BCG level 3. The combination decision rules for BCG level 3 required that all rules 1–4 must be accepted or rule 5 (% live *Orbicella* cover (DEMO)) was true (Table 3). At this site only two rules had a MemV = 1.00: LPI coral species > 4 (3–5) species was true with a MV = 5 and # non-tolerant coral spp. (LPI) > 2 (1–3) species with a MV = 4. The rule for % unproductive cover (LPI) < 30 (40–20) % was partially true with a MV = 32 and MemV = 0.40 (Table 4). The last rule considered for inclusion in BCG level 3 was % coral cover (LPI) > 20 (15–25) % that had a MV = 10 outside of the membership function with a MemV = 0.00. As such, the first four rules with MemV > 0.00 were not true for this site. Although the BCG level 3 combination rule 5 contains an optional rule that can override the failure of the rules 1–4, % live *Orbicella* (DEMO) however, the MV = 7 was outside the MemV = 0.00 for this optional rule, so the site cannot be assigned to BCG level 3. Next, the rules for BCG level 4 were considered. To assign the site to BCG level 4, three of the seven rules were required to be true. The highest MemVs for this site were 0.90 for % unproductive cover (LPI) (Rule 7), 0.60 for % non-tolerant coral cover (LPI) (Rule 2), and 0.54 for live coral cover 3D (DEMO) (Rule 3) (Table 4). All the other BCG level 4 rules had MemV = 0.00, except % live *Orbicella* (DEMO) for which MemV = 0.01 (Rule 4). The lowest value of the third nonzero membership value (Rule 3, 0.54) determined whether the site could be assigned to BCG level 4. The combination rule membership value for BCG level 4 was equal to 0.54. To determine whether the site should be assigned to BCG level 4 or 5, the MemVs for each of the three rules in BCG level 5 were considered. All the BCG rules for level 5 were true with MemV = 1.00. The membership value for assigning the BCG level cannot be > 1.00 by following fuzzy logic applications. The membership function value for the site belonging to BCG level 4 is 0.54 and the MemV for BCG level 5 is 1.00–0.54 = 0.46. For any site, if partial MemV for two BCG levels were within 0.10 of each other, then the model prediction was a tie between the two levels. The fore reef in St. Croix was considered a BCG level 4–5 tie.

## 5. Discussion

The numeric BCG model using coral reef benthic assemblages is one of two BCG numeric models for coral reefs that have been developed. This model can assist managers and other decision makers to protect and manage coral reef ecosystems, either individually or in conjunction with the coral reef fish BCG model (Bradley et al., 2020). Experts developed a sound and robust quantitative BCG model using benthic assemblages that primarily focused on scleractinian or hard-coral condition metrics. The numeric decision rules effectively built upon the narrative BCG model (Santavy et al., in review) using numeric metrics that were calibrated and validated using coral reef bioassessment data from Puerto Rico and USVI. This numeric model can be used as a tool to interpret the results of coral reef condition assessments and the cumulative biotic response to varying levels of anthropogenic stress while helping to inform management decisions.

The foundation of many environmental protection programs for local to national jurisdictions are water quality standards designed to protect, conserve, and restore aquatic life. One challenge for developing numeric or quantitative goals to implement policy and laws such as the US CWA include interpretation of narrative statements. It can be difficult to translate phrases such as “natural”, “degraded”, “balanced”, and “biological integrity” into numeric values that effectively represent terms found in policies and statutes (Paul et al., 2020). Quantitative models and rules can be used to clarify where narrative language might be subjective, and goals are vague. Quantitative decision rules are more defensible when regulatory proposals or decisions are challenged, providing objective metrics for decision support to identify important components for biological structure (e.g., biological integrity, biodiversity, etc.) and function (e.g., recruitment, productivity, reproduction, growth, etc.) throughout the coral reef.

There is an inherent chance for bias in a model that is based on expert judgment. To minimize this risk, we applied a modified Delphi-approach for expert-judgment modeling coupled with mathematical fuzzy set theory (Nair et al., 2011; Torres and Nieto 2006). The Delphi approach assures that the first round of BCG scores and revisions were kept anonymous prior to panel discussions. The BCG process allows each panel member to make individual judgments on the ecological significance of changes in the benthic assemblages, explain their logic, and only then, the panel comes to consensus on a set of quantitative decision rules for assigning sites to BCG levels through an iterative process (EPA 2016). Model decision rules developed by expert knowledge and judgment can reduce areas of ambiguity (e.g., what is expected at a site, what could be gained or lost from different management scenarios) and prevent eclipsing (e.g., loss of an ecologically critical indicator through averaging of multiple metrics) compared to statistical models derived solely from empirical data (Gerritsen et al., 2017).

To ensure credibility and improve validity for the Delphi and mathematical fuzzy set theory approaches, the expert panelists were intentionally selected to represent a broad base of expertise, experience, and institutional perspectives. Fifteen experts provided a wide and deep breadth of knowledge in coral reef ecology and taxonomy from a broad range of affiliations. The expert panel shared extensive cumulative experience that provided a robust understanding of the composition and function of historic reef communities, and personal observations of changes to reef communities prior to their exposure to widespread and significant anthropogenic stressors. This provided a retrospective view of BCG levels 1 and 2 and avoided incorrectly defining these levels based solely on present day condition of sites that exist in mostly degraded conditions. Finally, the panel included expertise in different and widely accepted methodological and analytical approaches for development of a robust and broadly applicable model. Strong and systematic facilitation was followed so that no single expert dominated panel deliberations. The 15 panelists evaluated 84 sites along a gradient of anthropogenic disturbance for a total of 544 individual ratings.

The availability of existing data representing a range of coral reef conditions was a particular challenge to calibrate the BCG model. Limited monitoring data are available to appropriately assess coral reef condition on a broad regional scale required for BCG model development. Most state, territorial and regional programs have no or very limited reef bioassessment programs. Data are available for many fewer reef sites collocated with water -quality monitoring, unlike many freshwater systems that have standardized water -quality and benthic -monitoring protocols used in survey programs assessed by states and regions often in operation for over 40 years. The availability of abundant, long-term data for freshwater systems enabled development of robust quantitative BCG models. Conversely, many coral reef bioassessments are small studies targeting specific locations or addressing very local research needs. To address this limitation, NOAA’s NCRMP has implemented nationally coordinated, continuous, and standardized coastal ocean assessments to develop biological status and indicators for priority U.S. coral reef areas (NOAA 2014). These data have been collected since 2013 in both the Western Atlantic and Pacific Islands and were recognized by the expert panel as the one of the most comprehensive dataset collections for coral condition in the US Caribbean Territories and the most suitable dataset to derive numeric BCG rules.

An important objective for the model was each numeric decision rule had discrete values and ranges that can be precisely written into computer algorithms or applied by practitioners to obtain reproducible BCG condition level assignments. The numeric BCG benthic model was accurate in matching the expert-derived BCG level. The numeric model predicted the panel-derived BCG median level for 84% of calibration sites and 89% of validation sites. These values are comparable to the model performances of other existing freshwater BCG quantitative models (Gerritsen et al., 2017; Paul et al., 2020). The numeric BCG model for coral reef fish also had comparable range where the model matched 92% calibration and 82% validation of the fish sites (Bradley et al., 2020).

**Fig. 9.**
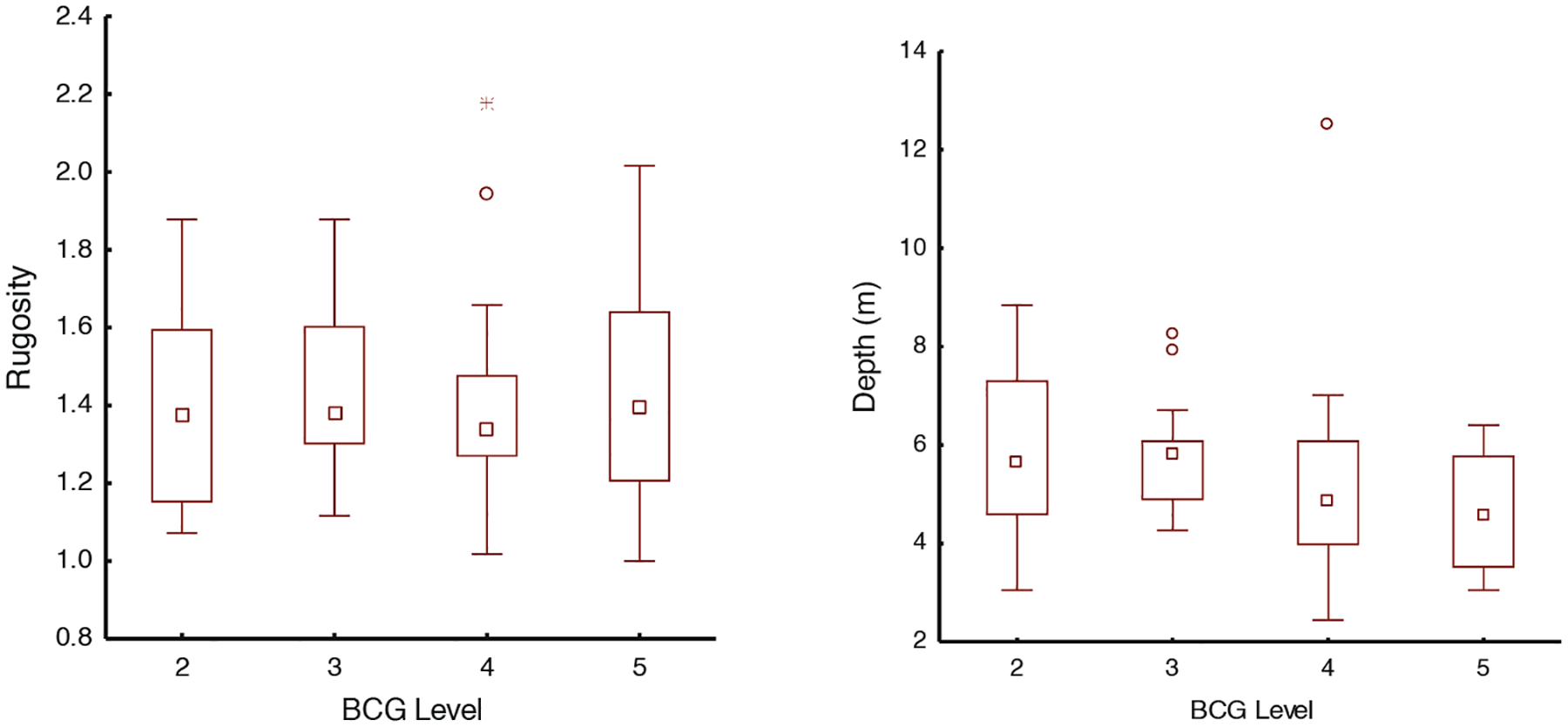
Box-and-whisker plots for additional benthic BCG metric values considered by the expert panel for developing quantitative rules for BCG levels. Rugosity data did not support the narrative rules and were not used in developing the model. Depth showed trends that were not related to either metric. Squares in boxes are medians, boxes are interquartile range (IQR), whiskers are to 1.5 × IQR, circles are outliers up to 3 IQR, and crosses show extreme values > 3 IQR.

Each narrative rule was tested to ensure that it was supported with empirical data used to develop the numeric (quantitative) BCG model. When the metric for the numeric rule was examined in the boxplot and did not support the narrative rule, the narrative rule was not further developed into a numeric model rule. For example, the proposed numeric rule and metric for rugosity were not substantiated by the NCRMP data, so the panel subsequently withdrew the rugosity metric rules (Fig. 9a). The expert panel proposed narrative rules for the BCG model expected high coral rugosity to indicate natural, high quality coral reef conditions and expected relative rugosity values to continue to decline with degraded coral reef conditions. Coral reef architectural complexity, measured as rugosity, has the capacity to provide the foundational taxa that highly influence the structure, function, and stability of the reef ecosystems (Alvarez-Filip et al., 2011). Alternatively, studies show decreases in coral reef condition and resilience are correlated with declines in rugosity values; increased anthropogenic stressors, including climate change, will cause greater reef degradation and coral mortality (Bozec et al., 2015). The most likely explanation was that different data sets were used to develop the narrative and numeric decision rules and more importantly each used different methods to define rugosity. For the narrative rules, rugosity was measured by the chain-draping method (Rogers et al., 1994) contained in the EPA dataset (Santavy et al., in review). The numeric rules employed the NCRMP dataset that used a different measure for surface complexity developed by the NOAA NCRMP program. The numeric rules were formulated using a newly developed microheterogeneity surface measurement (Brandt et al., 2009) to define a finer scale rugosity. The data from this new metric was not useful to discriminate rugosity among the BCG levels during model development. The panel discussed the merits and disadvantages of how to handle this situation. They agreed that a statistically sound bioassessment protocol might include studies that compare both methods for measuring rugosity and strongly recommended that monitoring of multiple transects should be made within a single location instead of just a single measurement as found in the dataset used. Depth was not related to BCG level assignment (Fig. 9b).

While the numeric BCG model for benthic assemblages was developed using data derived from fore reef environments off Puerto Rico and USVI, the rules provided in the numeric BCG model can provide a template to initiate development of numeric BCG models for coral reefs in other locations. This model can provide significant insight and underpin other efforts to assess coral reef condition using the BCG framework for other applications and locations. The model performance is comparable in accuracy and precision to other numeric BCG models developed for many different freshwater systems and locations currently used by states, tribes, and territories. The numeric BCG model for coral reef fish developed from Puerto Rico and USVI has been successfully tested for use in the Florida Keys and Dry Tortugas, in south Florida (Bradley et al., 2020). The benthic model has not been evaluated in other locations. Transference of the model would require testing and refining the decision rules using region-specific monitoring data; experts to recommend comparable functional groups in the new habitat or location that have decision rules devised for fore reefs in the US Caribbean territories; and vetting by regional experts. This numeric BCG model can provide a foundation to expand or modify the decision rules and metrics to accommodate more coral reef types, benthic assemblages, and other locations and regions for future evaluations.

This first-generation BCG model for coral-reef benthic assemblages identifies the structural and functional groups that maintain ecological integrity in environmental habitats or niches. The benthic model could be transferred to other locations or regions by identifying the structural and functional aspects of the model in other reef systems. Experts within a new locale can associate the appropriate species in the region to satisfy that role.

The numeric benthic BCG model developed here can be applied directly to coral reefs in PR and USVI and is readily adaptable to other locations. It is adaptable to incorporate scientific advancements in reef survey methods, as BCGs are developed as an iterative process that enables the narrative and numeric models to be updated when new information becomes available. Future research could support monitoring and assessment programs that implement new metrics, advance methodologies for measuring critical endpoints, and even might be flexible for more simplified screening-level assessments. Following implementation of the coral reef model by natural resource programs, increased monitoring efforts could further inform this model. The numeric BCG model using benthic assemblages is appropriate for adaptive environmental assessment and management applications and supports biological criteria development. The powerful assessment tool can be used to establish coral reef condition to protect habitats with high biological integrity, evaluate and communicate present or altering conditions, and develop restoration goals for managers, communities, and other interested beneficiaries.

## Supplementary Material

Supplementary data 5.

Supplementary data 4.

Supplementary data 6.

Supplementary data 3.

Supplementary data 2.

Supplementary data 1.

## Figures and Tables

**Table 1:** Benthic assemblages and substrate type categories assessed using the line point intercept method. (From NOAA NCRMP protocols 2014; Santavy et al. 2012).

Assemblage or Substrate Type	Subgroup	Species Grouping
Algal Groups	Microalgae	Diatoms/Cyanobacteria
	Macroalgae Fleshy	*Dictyota* spp. *Lobophora* spp. Other Fleshy spp.
	Macroalgae Calcareous	*Halimeda* spp. *Peysonnellia* Other Calcareous spp.
	Crustose Coralline Algae	*Rhodophyta crust. spp*. Other species
	Turf Algae	Turf Algae with no sediment Turf Algae with sediment
Submerged Aquatic Vegetation (SAV)	SAVs, Seagrasses	
Hard Coral species	Scleractinian and Hydrozoan Corals	Individual coral species recorded
Substrate Type	Bare *Cliona* spp.	Hard, Soft or Rubble Boring sponges^1^
Sponges	Other taxa	All others
Octocorals	Encrusting Gorgonians Gorgonacea plumes/rods/whips	
Zooanthids	*Palythoa* spp.	
Other species		

1 Poriferid sponges that secrete an acid that dissolves CaCO_3_ skeletons usually hard corals or shells. The sponge bores into the skeleton or shell and drills tunnels in the calcium matrix. Usually, the sponge genus *Cliona* will cause death of coral tissue.

**Table 2:** Number of sites assigned to BCG condition levels for calibration of model categorized by location, depth, and sampling method. Nine sites with no coral demographic (DEMO) data were not used for rule making. Total number of sites used is 57.

		3	4	5	6	No. sites	Grand total sites
Island	St. Thomas/St. John	16	10	2		28	
	St. Croix	3	11	3		17	
	Puerto Rico	0	13	8		21	66
Depth	Shallow (<40′)	2	12	11		25	
	Deep (>40′)	17	22	2		41	66
Method	LPI^1^ and DEMO^2^	17	28	12		57	
	LPI only	2	6	1		9	66
No. sites		19	34	13	0		66

1 Line Point Intercept survey method.

2 Demographic assessment of coral colonies survey method.

**Table 3:** BCG numeric model rules for coral reef benthic assemblages, showing the BCG level narrative and corresponding quantitative rules and combinations.

	BCG Metrics	Narrative Rules	Quantitative Rules
BCG level 3	1. Percent Live Coral Cover (LPI)	Moderate coral cover	> 20% (15–25)
	2. Total Coral Richness (LPI)	Moderate coral richness	> 4 species (3–5)
	3. Non-tolerant Coral Richness (LPI)	Non-tolerant BCG Attribute I, II, III, IV taxa are present	> 2 species (1–3)^a^
	4. Bare Substrate and Turf with Sediment Cover (LPI)	Minimal presence of unproductive and sedimented substrate	< 30% (20–40)
	5. Percent Live *Orbicella* cover (DEMO)	Moderate presence of *Orbicella* colonies	> 20% (15–25)
	Level 3 Combination: Minimum of first 4 rules or the *Orbicella* rule^b^		
BCG level 4	1. Percent Coral Cover (LPI)	Low to moderate total coral cover	>15% (10–20)
	2. Non-tolerant Coral Cover (LPI)	Low to moderate non-tolerant BCG Attribute I, II, III, IV cover	> 5% (0–10)^a^
	3. Live Coral Cover (DEMO)	Low to moderate total coral cover (based on surface area 3-D)	> 2000 cm^2^/m^2^ (1000–3000)
	4. Percent live *Orbicella* cover (DEMO)	*Orbicella* present, though sparse	> 2.5% (0–5)
	5. Percent *Orbicella* cover (LPI)	*Orbicella* present, though sparse	> 2.5% (0–5)
	6. Density of medium or large colonies (DEMO)	Medium size colonies (max D > 20 cm) present in the transect	> 7.5 colonies (5–10)
	7. Bare Substrate and Turf with Sediment Cover (LPI)	Moderate presence of unproductive and sedimented substrate	< 40% (30–50)^c^
	Level 4 Combination: Minimum of the three highest membership values^d^		
BCG level 5	1. Percent Coral Cover (LPI)	At least some living coral	> 5% (2–8)^e^
	2. Density of Colonies (DEMO)	At least some living coral	> 1 colony/m^2^ (0–2)
	3. Non-tolerant coral spp. richness (DEMO)	Attribute I, II, III, or IV taxa are present	> 1 species (0–2)
	Level 5 Combination: Minimum of the two highest membership values		
BCG level 6		Absence of colonies; those present are small; only tolerant species; little or no tissue	

a) Attribute I taxa were included because, though they are not specifically non-tolerant, they are in some way specialists, endemic, or long-living.

b) Live 2D cover of *Orbicella* does not need to be high for a reef to be level 3 (if *Orbicella* cover is < 20%, the minimum of the other rules is the predicted membership of level 4). However, if *Orbicella* cover is > 20%, then the *Orbicella* rule alone can override the minimum of the other four rules.

c) The expert panel expressed that a rule regarding algae should be applied in Level 4. The rule on bare substrate and turf algae with sediment was added compared to the previous model draft.

d) The expert panel suggested that three rules should be met instead of only two that were required in the previous model draft. This rule on its own would result in additional model errors, but when also adding the bare substrate and turf with sediment rule, no additional model errors resulted. The level 4 rule thresholds were established to identify possible level 4 conditions, rather than to screen out level 5 conditions, so only a few indications are required.

e) Experts suggested raising the % LPI cover threshold to 5% instead of the previous threshold of 2%. Raising the LPI % cover threshold resulted in 5 errors at level 5 (predicting level 6 conditions for this rule)

**Table 4:** Application of model to assign BCG condition level to a study site, using membership formulas for each decision rule and evaluating consolidated membership values for all rules at each BCG level.

BCG level	Variable name	BCG level rule	Membership Formula	Metric Value (MV)	Membership Value (MemV)	Required number rules for level inclusion	Rule membership value
3	% coral cover (LPI)	1. LPI % live coral cover	> 20 (15–25) %	10	0.00	Min. value of first 4 rules	0.00
	# coral spp. (LPI)	2. LPI coral species	> 4 (3–5) species	5	1.00	Or	
	# non-tolerant coral spp. (LPI)	3. LPI Attribute II, III, IV species	> 2 (1–3) species	4	1.00	Optional Rule 5	0.00
	% unproductive cover (LPI)	4. Bare Substrate and Turf with Sediment	< 30 (40–20) %	32	0.40		
	% live *Orbicella* (DEMO)	5. Live Cover of *Orbicella* (optional)	> 20 (15–25) %	7	0.00	**Level 3 membership**	**0.00**
4	% coral cover (LPI)	1. % LPI coral cover > 15	> 15 (10–20) %	10	0.00	Min value of 3 rules	0.54
	% non-tolerant coral cover (LPI)	2. % LPI Att 2,3,4 cover > 5	> 5 (0–10) %	6	0.60		
	live coral cover 3D (DEMO)	3. 3D Live DEMO coral cover > 2000	> 2000 (1000–3000)	2075	0.54		
	% *Orbicella* cover (DEMO)	4. 2D Live cover of *Orbicella >* 250	> 2.5 (0–5) %	7	0.01		
	density med-large colonies (DEMO)	5. No. DEMO colonies > 20 cm diameter > 7	> 7.5 (5–10) colonies	3	0.00		
	% *Orbicella cover (LPI)*	6. % LPI *Orbicella* cover > 2	> 2.5 (0–5) %	0	0.00		
	% unproductive cover (LPI)	7. Turf and bare sediment < 40	< 40 (30–50) %	32	0.90	**Level 4 membership**	**0.54**
5	% coral cover (LPI)	1. % LPI coral cover	> 5 (2–8) %	10	1.00	Min. value of 2 rules	1.00
	colony density (DEMO)	2. Density of DEMO colonies	> 1 (0–2) colonies	6	1.00		
	# non-tolerant coral spp. (DEMO)	3. DEMO coral species	> 1 (0–2) species	8	1.00	**Level 5 membership**	**0.46**=1.00–0.54

## References

[R1] Alvarez-Filip L, Dulvy NK, Côté IM, Watkinson AR, Gill JA, 2011. Coral identity underpins architectural complexity on Caribbean reefs. Ecol. Appl 21, 2223–2231. 21939056 10.1890/10-1563.1

[R2] Bozec YM, Alvarez-Filip L, Mumby PJ, 2015. The dynamics of architectural complexity on coral reefs under climate change. Glob. Change Biol 21, 223–235. 10.1111/gcb.12698 25099220

[R3] Brandt ME, Zurcher N, Acosta A, Ault JS, Bohnsack JA, Feeley MW, Harper DE, Hunt JH, Kellison T, McClellan DB, Patterson ME, Smith SG 2009. A cooperative multi-agency reef fish monitoring protocol for the Florida Keys coral reef ecosystem. Natural Resource Report NPS/SFCN/NRR-2009/150, Fort Collins, Colorado, National Park Service.

[R4] Bradley P, Jessup B, Pittman SJ, Jeffrey CFJ, Ault JS, Carrubba L, Lilyestrom C, Appeldoorn R, McField M, Schärer MT, Santavy DL, Wojtenko I, Smith T, Garcia G, Huertas E, Murry B, Walker BK, Ramos A, Gerritsen J, Jackson SK, 2020. Using reef fish as biocriteria to protect Caribbean coral reef ecosystems. Mar. Pollut. Bull 159, 111287. 10.1016/j.marpolbul.2020.111387 PMC8717739 32827871

[R5] Costa BM, Bauer LJ, Battista TA, Mueller PW, Monaco ME 2009. Moderate-Depth Benthic Habitats of St. John, U.S. Virgin Islands. NOAA Technical Memorandum NOS NCCOS 105. NCCOS Center for Coastal Monitoring and Assessment Biogeography Branch Silver Spring, MD.

[R6] Costa BM, Kendall MS, Edwards K, Kagesten G, Battista TA, 2013. Benthic Habitats of Fish Bay. NOAA Technical Memorandum NOS NCCOS, Coral Bay and the St Thomas East End Reserve, p. 175.

[R7] Darling ES, McClanahan TR, Maina J, Gurney GG, Graham NAJ, Januchowski-Hartley F, Cinner JE, Mora C, Hicks CC, Maire E, Puotinen M, 2019. Social–environmental drivers inform strategic management of coral reefs in the Anthropocene. Nat. Ecol. Evol 3, 1341–1350. 31406279 10.1038/s41559-019-0953-8

[R8] Davies SP, Jackson SK, 2006. The Biological Condition Gradient: a descriptive model for interpreting change in aquatic ecosystems. Ecol. Appl 16, 1251–1266. 16937795 10.1890/1051-0761(2006)016[1251:tbcgad]2.0.co;2

[R9] Droesen WJ, 1996. Formalization of ecohydrological expert knowledge applying fuzzy techniques. Ecol. Model 85, 75–81.

[R10] Fabricius KE, 2005. Effects of terrestrial runoff on the ecology of corals and coral reefs: review and synthesis. Mar. Pollut. Bull 50, 125–146. 15737355 10.1016/j.marpolbul.2004.11.028

[R11] Fisher WS, Vivian DN, Campbell J, LoBue C, Hemmer RL, Wilkinson S, Harris P, Santavy DL, Parsons M, Bradley P, Humphrey A, Oliver LM, Harwell L, 2019. Biological Assessment of Coral Reefs in Southern Puerto Rico: supporting coral reef protection under the U.S. Clean Water Act. Coastal Manage 47, 429–452. 10.1080/08920753.2019.1641039 PMC6781237 31595103

[R12] Gerritsen J, Bouchard RW Jr, Zheng L, Leppo EW, Yoder CO, 2017. Calibration of the biological condition gradient in Minnesota streams: a quantitative expert-based decision system. Freshwater Sci 36, 427–451.

[R13] Harborne AR, Rogers A, Bozec Y, Mumby PJ, 2017. Multiple Stressors and the Functioning of Coral Reefs. Annual Review of Marine Science 9, 445–468. 10.1146/annurev-marine-010816-060551 27575738

[R14] Hill J, Wilkinson CE, 2004. Methods for ecological monitoring of coral reefs Australian Institute of Marine Science, Townsville, p. 117.

[R15] Hausmann S, Charles DF, Gerritsen J, Belton TJ, 2016. A diatom-based biological condition gradient (BCG) approach for assessing impairment and developing nutrient criteria for streams. Sci. Total Environ 562, 914–927. 27128024 10.1016/j.scitotenv.2016.03.173

[R16] Hernández R, Sherman C, Weil E, Yoshioka P, 2009. Spatial and temporal patterns in reef sediment accumulation and composition, southwestern insular shelf of Puerto Rico. Carib. J. Sci 45, 138–150.

[R17] Hughes TP, Kerry T, Simpson T, 2018. Large-scale bleaching of corals on the Great Barrier Reef. Ecology 99, 501. 29155453 10.1002/ecy.2092

[R18] Hughes TP, Rodrigues MJ, Bellwood DR, Ceccarelli D, Hoegh-Guldberg O, McCook L, Moltschaniwskyj N, Pratchett MS, Steneck RS, Willis B, 2007. Phase shifts, herbivory, and the resilience of coral reefs to climate change. Curr. Biol 17, 360–365. 17291763 10.1016/j.cub.2006.12.049

[R19] Jokiel PL, Rodgers KS, Brown EK, Kenyon JC, Aeby G, Smith WR, Farrell F, 2015. Comparison of methods used to estimate coral cover in the Hawaiian Islands. Peer Journal 3, e954. 10.7717/peerj.954 PMC4435506 26020009

[R20] Lang JC 2003. Caveats for the AGRRA “Initial Results Volume.” In: Lang JC (ed.), Status of Coral Reefs in the western Atlantic: Results of initial Surveys, Atlantic and Gulf Rapid Reef Assessment (AGRRA) Program Atoll Research Bulletin 496.

[R21] Loya Y, 1976. Effects of water turbidity and sedimentation on the community structure of Puerto Rican corals. Bull. Mar. Sci 26, 450–466.

[R22] Moberg F, Folke C, 1999. Ecological goods and services of coral reef ecosystems. Ecol. Econ 29, 215–233.

[R23] Nair R, Aggarwal R, Khanna D, 2011. Methods of formal consensus in classification/diagnostic criteria and guideline development. Semin. Arthritis Rheum 41, 95–105. 21420149 10.1016/j.semarthrit.2010.12.001 PMC3131416

[R24] NOAA. 2015. Recovery Plan for Elkhorn (*Acropora palmata*) and Staghorn (*A. cervicornis*) Corals. Prepared by the Acropora Recovery Team for the National Marine Fisheries Service, Silver Spring, Maryland.

[R25] NOAA Coral Program, 2014. National Coral Reef Monitoring Plan. Silver Spring, MD, NOAA. Coral Reef Conservation Program. 40 pp.

[R26] NOAA NCRMP 2013 USVI. 2013. NCRMP assessment data: Assessment of coral reef benthic communities in the U.S. Virgin Islands; https://data.nodc.noaa.gov/cgi-bin/iso?id=gov.noaa.nodc:NCRMP-Benthic-USVI, (last accessed March 2018).

[R27] NOAA NCRMP 2014 Puerto Rico. 2014. NCRMP assessment data: Assessment of coral reef benthic communities in Puerto Rico; https://data.nodc.noaa.gov/cgi-bin/iso?id=gov.noaa.nodc:NCRMP-Benthic-PR (last accessed March 2018).

[R28] Oliver LM, Fisher WS, Fore L, Smith A, Bradley P, 2018. Assessing land use, sedimentation, and water quality stressors as predictors of coral reef condition in St. Thomas, U.S. Virgin Islands. Environ. Monitor. Assess 190, 213–228. 10.1007/s10661-018-6562-1 PMC6251406 29536196

[R29] Paul MJ, Jessup B, Brown LR, Carter JL, Cantonati M, Charles DF, Gerritsen J, Herbst DB, Stancheva R, Howard J, Isham B, 2020. Characterizing benthic macroinvertebrate and algal biological condition gradient models for California wadeable Streams, USA. Ecol. Ind 117, 106618.

[R30] Pollock FJ, Lamb JB, Field SN, Heron SF, Schaffelke B, Shedrawi G, Bourne DG, Willis BL, 2014. Sediment and turbidity associated with offshore dredging increase coral disease prevalence on nearby reefs. PLoS ONE 9 (7), e102498. 25029525 10.1371/journal.pone.0102498 PMC4100925

[R31] Principe PP, Bradley P, Yee S, Fisher WS, Johnson E, Allen P, Campbell D 2012. Quantifying coral reef ecosystem services. EPA/ 600/R-11/206. U.S. Environmental Protection Agency, Office of Research and Development, Research Triangle Park.

[R32] Reguero BG, Storlazzi CD, Gibbs AE, Shope JB, Cole AD, Cumming KA, Beck MW, 2021. The value of US coral reefs for flood risk reduction. Nat. Sustainability 4, 688–698.

[R33] Rogers CS, Garrison G, Grober R, Hillis ZM, Franke MA, 1994. Coral reef monitoring manual for the Caribbean and Western Atlantic. US National Park Service, St. John, US Virgin Islands.

[R34] Santavy DL, Fisher WS, Campbell JG, Quarles RL 2012. Field manual for coral reef assessments U.S. Environmental Protection Agency, Office of Research and Development, Gulf Ecology Division, Gulf Breeze, FL. EPA/ 600/R-12/029. April 2012.

[R35] Santavy DL, Bradley P, Gerritsen J, Oliver L, 2016. The Biological Condition Gradient, a Tool used for Describing the Condition of US Coral Reef Ecosystems. Proceedings of 13th International Coral Reef Symposium 557–568.

[R36] Santavy DL, Horstmann CL, Sharpe LM, Yee SH, Ringold P, 2021. What is it about coral reefs? Translation of ecosystem goods and services relevant to people and their well-being. Ecosphere 12 (8), e03639. 10.1002/ecs2.3639. PMC8686212 34938591

[R37] Santavy DL, Jackson SK, Jessup B, Gerritsen J, Rogers C, Fisher WS, Weil E, Szmant A, Cuevas Miranda D, Walker BK, Jeffrey C, Bradley P, Ballantine D, Roberson L, Ruiz Torres H, Todd B, Smith T, Clark R, Diaz E, Bauźa-Ortega J, Horstmann C, Raimondo S In review. A Biological Condition Gradient for Caribbean Coral Reefs: Part I. Coral Narrative Rules. Ecological Indicators. ECOLIND-21144 10.1016/j.ecolind.2022.108805 PMC9904394 36761828

[R38] Shumchenia EJ, Pelletier MC, Cicchetti G, Davies S, Pesch CE, Deacutis CF, Pryor M, 2015. A biological condition gradient model for historical assessment of estuarine habitat structure. Environ. Manage 55, 143–158. 25387456 10.1007/s00267-014-0401-0

[R39] Smith TB, Nemeth RS, Blondeau J, Calnan JM, Kadison E, Herzlieb S, 2008. Assessing coral reef health across onshore to offshore stress gradients. Mar. Pollut. Bull 56, 1983–1991. 18834601 10.1016/j.marpolbul.2008.08.015

[R40] Storlazzi CD, Reguero BG, Cole AD, Lowe E, Shope JB, Gibbs AE, Nickel BA, McCall RT, van Dongeren AR, Beck MW 2019, Rigorously valuing the role of U. S. coral reefs in coastal hazard risk reduction: U.S. Geological Survey Open-File Report 2019–1027, 42 p., 10.3133/ofr20191027.

[R41] Torres A, Nieto JJ, 2006. Fuzzy logic in medicine and bioinformatics. J. Biomed. Biotechnol 2006 (91908), 2021. 10.1155/JBB/2006/91908 (Last accessed September. PMC1559939 16883057

[R42] US EPA. 2016. A Practitioner’s Guide to the Biological Condition Gradient: A Framework to Describe Incremental Change in Aquatic Ecosystems US Environmental Protection Agency, Washington, DC. EPA/842/R-16/001.

[R43] van Beukering P, Brander L, Zanten BV, Verbrugge E, Lems K 2011. The economic value of the coral reef ecosystems of the United States Virgin Islands. Report R-11/06 IVM Institute for Environmental Studies, Amsterdam.

[R44] Wilkinson CR 2008. Status of Caribbean Coral Reefs After Bleaching and Hurricanes in 2005 Global Coral Reef Monitoring Network, and Reef and Rainforest Research Centre, Townsville, Australia.

[R45] Williams GJ, Graham NAJ, Jouffray JB, Norström AV, Nyström M, Gove JM, Heenan A, Wedding LM, 2019. Coral reef ecology in the Anthropocene. Funct. Ecol 33, 1014–1022.

[R46] Williams SM, Mumby PJ, Chollett I, Cortes J, 2015. Importance of differentiating *Orbicella* reefs from gorgonian plains for ecological assessments of Caribbean reefs. Mar. Ecol. Prog. Ser 530, 93–101.

[R47] Woodhead AJ, Hicks CC, Norström AV, Williams GJ, Graham NA, 2019. Coral reef ecosystem services in the Anthropocene. Funct. Ecol 33, 1023–1034.

[R48] Zadeh LA, 1965. Fuzzy sets. Inf. Control 8, 338–353.

[R49] Zadeh LA, 2008. Is there a need for fuzzy logic? Inf. Sci 178, 2751–2779.

