# Supplementary material for: A biological condition gradient for Caribbean coral reefs: Part II. Numeric rules using sessile benthic organisms
Source: Ecol Indic. Author manuscript; Available in PMC 2022 May 4. (PMC9067392; doi:10.1016/j.ecolind.2022.108576)
Supplement: Supplementary data 5. [file NIHMS1794197-supplement-Supplementary_data_5_.docx]

**Supplemental Information F**

**Summary statistics for all metrics in numeric model**

| Table F1: Summary statistics of metrics used in numeric model. Metric variable name highlighted in yellow. Metrics included in BCG level 2 conceptual description are highlighted in grey rows. Green highlights indicate metric used in numeric rule for specific BCG level. | | | | | | | | | | |
| --- | --- | --- | --- | --- | --- | --- | --- | --- | --- | --- |
| Description of Metric) | BCG level | Valid N | Mean | Median | Min  value | Max  value | Lower Quartile | Upper Quartile | Std.  Dev. | BCG rule |
| Percent Coral Cover (LPI) | 2 |  |  |  |  |  |  |  |  | Y |
| % coral cover (LPI) | 3 | 19 | 31.84 | 33 | 17 | 46 | 27 | 37 | 7.25 | Y |
|  | 4 | 34 | 16.32 | 16 | 4 | 37 | 11 | 20 | 7.07 | Y |
|  | 5 | 14 | 7.86 | 7 | 2 | 17 | 3 | 12 | 4.97 | Y |
| Total Coral Richness (LPI) | 3 | 19 | 6.79 | 6 | 4 | 10 | 5 | 8 | 2.07 | Y |
| # coral spp. (LPI) | 4 | 34 | 6.06 | 6 | 2 | 9 | 4 | 8 | 2.06 | N |
|  | 5 | 14 | 4.21 | 5 | 1 | 10 | 2 | 5 | 2.33 | N |
| Non-tolerant BCG Attribute I, II, III, IV taxa present (LPI) | 3 | 19 | 4 | 4 | 2 | 7 | 3 | 5 | 1.53 | Y |
| # non-tolerant coral spp. (LPI) | 4 | 34 | 2.97 | 3 | 0 | 6 | 2 | 4 | 1.31 | N |
|  | 5 | 13 | 1.92 | 2 | 0 | 7 | 0 | 2 | 1.98 | N |
| Presence of unproductive and sedimented cover (LPI) | 3 | 19 | 10.89 | 7 | 0 | 30 | 5 | 17 | 8.74 | Y |
| % unproductive cover (LPI) | 4 | 34 | 30.32 | 26.5 | 0 | 91 | 16 | 45 | 20.88 | Y |
| Percent Bare Substrate and Turf with Sediment Cover | 5 | 13 | 41.08 | 42 | 4 | 81 | 30 | 53 | 23.22 | N |
| Percent live *Orbicella* cover (DEMO) | 2 |  |  |  |  |  |  |  |  | Y |
| % live *Orbicella* (DEMO) | 3 | 17 | 24.09 | 26.42 | 14 | 61.67 | 9.99 | 30.1 | 16.44 | Y |
| Live coral surface area (2D) | 4 | 28 | 7.59 | 4 | 0 | 38.93 | 0.99 | 13.48 | 8.82 | Y |
|  | 5 | 13 | 0.7 | 0 | 0 | 6.37 | 0 | 0.23 | 1.79 | N |
|  |  |  |  |  |  |  |  |  |  |  |
| Description of Metric) | BCG level | Valid N | Mean | Median | Min  value | Max  value | Lower Quartile | Upper Quartile | Std.  Dev. | BCG rule |
| Live coral cover (DEMO) (cm^2^/cm^2^) | 3 | 17 | 5494 | 5552 | 1590 | 11701 | 3579 | 6794 | 2698 | N |
| live coral cover 3D (DEMO) | 4 | 28 | 3961 | 2840 | 246 | 23546 | 1374 | 4489 | 4775 | Y |
| Low to moderate total coral cover (surface area 3-D) | 5 | 13 | 1120 | 889 | 267 | 4162 | 588 | 1258 | 993 | N |
| Density medium/ large colonies (DEMO) | 3 | 17 | 23.6 | 28 | 2 | 37 | 17 | 31 | 10.42 | N |
| density med-large colonies (DEMO) | 4 | 28 | 11 | 9 | 1 | 29 | 5 | 14 | 7.63 | Y |
| Colony max diameter > 20cm | 5 | 12 | 3.7 | 3.5 | 0 | 8 | 1.5 | 5.5 | 2.42 | N |
| Percent *Orbicella* cover (LPI) | 3 | 19 | 20.2 | 24 | 1 | 38 | 8 | 28 | 10.49 | N |
| % *Orbicella* cover (LPI) | 4 | 34 | 5.8 | 5 | 0 | 24 | 1 | 9 | 5.73 | Y |
|  | 5 | 13 | 1.2 | 0 | 0 | 8 | 0 | 1 | 2.24 | N |
| Percent non-tolerant BCG Attribute I, II, III, IV taxa (LPI) | 3 | 19 | 24.6 | 26 | 3 | 42 | 18 | 31 | 9.11 | N |
| % non-tolerant coral cover (LPI) | 4 | 34 | 9.4 | 8 | 0 | 24 | 4 | 13 | 5.98 | Y |
|  | 5 | 13 | 3.5 | 2 | 0 | 12 | 0 | 5 | 3.91 | N |
| Density of Colonies #/m2 (DEMO) | 3 | 17 | 7.18 | 6.6 | 3.4 | 13.9 | 5.5 | 8.1 | 2.63 | N |
| colony density (DEMO) | 4 | 28 | 6.05 | 6.1 | 2 | 12.2 | 4.1 | 7.9 | 2.61 | N |
|  | 5 | 12 | 2.88 | 2.1 | 1.3 | 5.2 | 1.7 | 4.5 | 1.45 | Y |
| Non-tolerant Coral Richness (DEMO) | 3 | 17 | 6.65 | 7 | 3 | 12 | 5 | 7 | 2.12 | N |
| # non-tolerant coral spp. (DEMO) | 4 | 28 | 6.43 | 6 | 2 | 12 | 4 | 9 | 2.86 | N |
| (Non-tolerant coral taxa Attribute I-IV taxa) | 5 | 12 | 3.58 | 4 | 0 | 8 | 2 | 5 | 2.54 | Y |
