# Supplementary material for: A biological condition gradient for Caribbean coral reefs: Part II. Numeric rules using sessile benthic organisms
Source: Ecol Indic. Author manuscript; Available in PMC 2022 May 4. (PMC9067392; doi:10.1016/j.ecolind.2022.108576)
Supplement: Supplementary data 4. [file NIHMS1794197-supplement-Supplementary_data_4_.docx]

**Supplemental Information D**

**Generic BCG attribute definitions and coral species assignments**

**to elevated temperature and sediment**

Ten BCG attributes are shown to be responsive to taxa structure and compositional changes when exposed to major anthropogenic stressors (Davies and Jackson 2006; US EPA 2016) (Table D1). During the workshops, experts assigned each Caribbean coral species to one of the first six BCG attributes (herein represented by Roman numerals). Only the first six attributes were assigned to coral species, as the information needed to inform attributes VII-X were either not fully developed for coral reef assemblages (BCG attributes VII and VIII) or applicable at the scale of coral reefs (BCG attributes IX and X).

| Table D1: General descriptions of the BCG attributes used to assign taxa into BCG attribute levels I-VI for scleractinian coral reef communities (adapted from US EPA 2016). | | |
| --- | --- | --- |
| Attribute Number | Attribute Name | Attribute Description |
| I | Historically documented, sensitive, long-lived, or regionally endemic taxa | Taxa known to have been supported according to historical, museum, or archeological records, or taxa with restricted distribution (occurring only in a locale as opposed to a region), often because of unique life-history requirements |
| II | Highly sensitive taxa | Taxa that are highly sensitive to pollution or anthropogenic disturbance; tend to occur in low numbers, and many are specialists for habitats and food type; the first to disappear with disturbance or pollution |
| III | Intermediate sensitive taxa | Common taxa that are ubiquitous and abundant in relatively undisturbed conditions but are sensitive to anthropogenic disturbance/pollution; have a broader range of tolerance than highly sensitive taxa |
| IV | Intermediate tolerant taxa | Ubiquitous and common taxa that can be found under almost any conditions, from undisturbed to highly stressed sites; broadly tolerant but often decline under extreme conditions |
| V | Tolerant taxa | Taxa that typically are uncommon and of low abundance in undisturbed conditions but increase in abundance in disturbed sites; opportunistic species able to exploit resources in disturbed sites; the last survivors |
| VI | Non-native or intentionally introduced species | Any species not native to the ecosystem |
| VII | Organism condition | Anomalies of the organisms; indicators of individual health (e.g., deformities, lesions, tumors) |
| VIII | Ecosystem function | Processes performed by ecosystems, including primary and secondary production, respiration, nutrient cycling, decomposition, their proportion/dominance, and what components of the system carry the dominant functions |
| IX | Spatial and temporal extent of detrimental effects | The spatial and temporal extent of cumulative adverse effects of stressors |
| X | Ecosystem connectance | Access or linkage (in space/time) to materials, locations, and conditions required for maintenance of interacting populations of aquatic life; the opposite of fragmentation |

Each coral species was first assigned to an attribute level I–V based on their sensitivity and tolerance to elevated sea water temperature and by their sensitivity to sediment. Sediment was used as a surrogate for land-based sources of pollutant runoff. Attribute VI was assigned to non-native or invasive species. If a species could not be confidently designated to a BCG attribute level from I-V by the panel no assignment was made. The experts attempted to assign 48 scleractinian and three hydrozoan hard coral species of the Western Atlantic to BCG attributes II–V, VI non-native taxa, or no assignment if the experts did not have sufficient experience or knowledge to assign taxon to an attribute. No species were assigned to BCG attribute I that were endemic and rare. BCG attributes II through V were assigned to species with increasing levels of tolerance to stress, with BCG attribute II highly sensitive and BCG attribute V highly tolerant.

| Table D2: Sensitivity and tolerance of scleractinian species to sediment and elevated sea temperature assigned to BCG attributes I-V by expert panel. I= rare, long-lived or endemic taxa; II = highly sensitive taxa; III = moderately sensitive; IV = intermediate, broadly tolerant; V = tolerant to pollution; VI = nonnative; X = insufficient information; (See Table D1 for generic definitions). | | | |
| --- | --- | --- | --- |
| BCG Attribute | Scientific Name | Sediment stressors | Heat Tolerance |
| II | *Isophyllia rigida* | II | II |
| II | *Isophyllia sinuosa* | II | II |
| III | *Helioseris cucullata* | III | III |
| III | *Acropora cervicornis* | III | III |
| III | *Agaricia lamarcki* | III | II |
| III | *Colpophyllia natans* | III | III |
| III | *Dendrogyra cylindrus* | III - IV | III |
| III | *Diploria labyrinthiformis* | III | III |
| III | *Eusmilia fastigiata* | III | III |
| III | *Madracis decactis* | II - IV | IV |
| III | *Millepora complanata* | III | II |
| IV | *Acropora prolifera* | IV | III |
| IV | *Cladocora arbuscula* | IV | IV |
| IV | *Meandrina jacksoni^1^* | IV | III |
| IV | *Mussa angulosa* | IV | II |
| IV | *Scolymia cubensis* | IV | IV |
| IV | *Scolymia lacera* | IV | IV |
| IV | *Acropora palmata* | IV | III |
| IV | *Agaricia agaricites* | IV | II |
| IV | *Agaricia humilis* | IV | II |
| IV | *Dichocoenia stokesii* | IV | III |
| IV | *Madracis auretenra^2^* | IV | III |
| IV | *Meandrina meandrites* | IV | III |
| IV | *Mycetophyllia aliciae* | IV | III |
| IV | *Mycetophyllia ferox* | IV | II - III |
| IV | *Orbicella annularis* | IV | II |
| IV | *Orbicella faveolata* | IV | II |
| IV | *Orbicella franksi* | IV | II |
| IV | *Porites furcata* | IV | IV - V |
| IV | *Porites porites* | IV | IV |
| V | *Favia fragum* | V | IV |
| V | *Manicina areolata* | V | V |
| V | *Oculina diffusa* | V | IV |
| V | *Montastraea cavernosa* | V | IV - V |
| V | *Porites astreoides* | V | V |
| V | *Porites divaricata* | V | IV |
| V | *Pseudodiploria clivosa* | V | IV |
| V | *Pseudodiploria strigosa* | V | IV |
| V | *Siderastrea radians* | V | V |
| V | *Siderastrea siderea* | V | IV |
| V | *Solenastrea bournoni* | V | IV |
| V | *Stephanocoenia intersepta* | V | IV |
| V | *Millepora alcicornis* | V | II |
| VI | *Tubastrea coccinea* |  |  |
| x | *Millepora squarrosa* | X | II |
| x | *Agaricia fragilis* | X | X |
| x | *Mycetophyllia daniana* | X | X |
| x | *Mycetophyllia lamarckiana* | X | X |
| x | *Porites branneri* | X |  |

^1^ Combined *M. jacksoni* with *M. meandrites* (Pinzón and Weil 2011)

^2^ Changed from *Madracis mirabilis* (Locke et al. 2007)

**References**

Davies, S.P., Jackson, S.K. 2006. The Biological Condition Gradient: a descriptive model for interpreting change in aquatic ecosystems. Ecological Applications 16: 1251–1266.

Locke, J.M., Weil, E., Coates, K.A. 2007 A newly documented species of *Madracis* (Scleractinia: Pocilloporidae) from the Caribbean. Proceedings of the Biological Society of Washington 120:214-226.

Pinzón, J.H. and Weil, E. 2011. Cryptic Species Within the Atlantic-Caribbean Genus *Meandrina* (Scleractinia): A Multidisciplinary Approach and Description of the New Species *Meandrina jacksoni*</I. Bulletin of Marine Science 87: 823-853.

US EPA. 2016. A Practitioner’s Guide to the Biological Condition Gradient: A Framework to Describe Incremental Change in Aquatic Ecosystems. US Environmental Protection Agency, Washington, DC. EPA/842/R-16/001.
