# Supplementary material for: A biological condition gradient for Caribbean coral reefs: Part II. Numeric rules using sessile benthic organisms
Source: Ecol Indic. Author manuscript; Available in PMC 2022 May 4. (PMC9067392; doi:10.1016/j.ecolind.2022.108576)
Supplement: Supplementary data 6. [file NIHMS1794197-supplement-Supplementary_data_6_.docx]

**Supplemental Information G**

**Excel worksheet template provided to experts**

**for calibration and validation of numeric model (LPI, DEMO)**

The Demographic (DEMO) Reef Coral Survey method was developed by the NOAA National Coral Reef Monitoring Program (NCRMP). Divers swim along a 10 m x 1 m belt transect in hard-bottom and coral reef habitats, recording information on species composition, density, size, abundance, and specific parameters of condition (% live vs. dead and bleaching) of non-juvenile scleractinian corals (> 4 cm maximum diameter), and of overall species diversity (all corals). These metrics provide information on two key reef characteristics coral and condition (Figure G1).

NOAA NCRMP employed the Line-Point Intercept (LPI) method to estimate the percent benthic coverage of ecologically important cover types (macroalgae, turf algae, crustose coralline algae, corals, sponges, sand/sediment, etc.). This method used points along a 25m transect to quantify each of the benthic organism or substrate types lying every 20 cm under the tape, a total of 100 points with tally of substrates and biota. Because the intervals were 100th of the transect length, each point constituted 1% of cover. This survey method provided information on many more species than just corals but is a quicker and less detailed survey method. Experts were shown these survey results in addition to the demographic survey metrics to make their BCG level ratings (Figure G2).


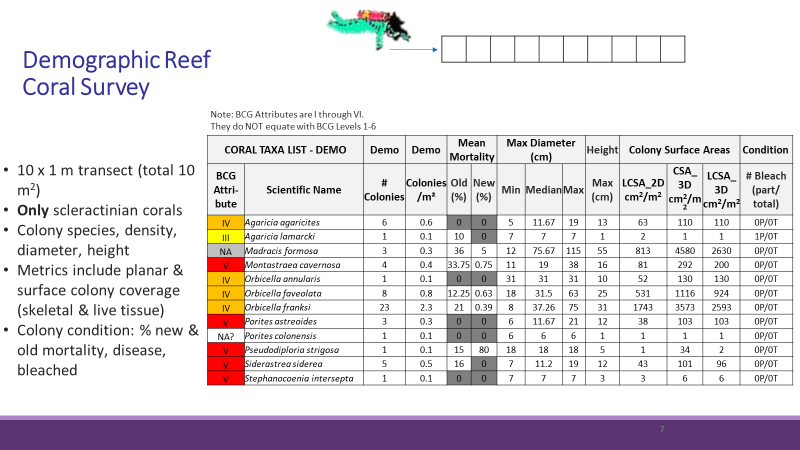


Figure G1: DEMO homework page that contains table given to expert panel to provide context for reef condition to decide their BCG level ratings.


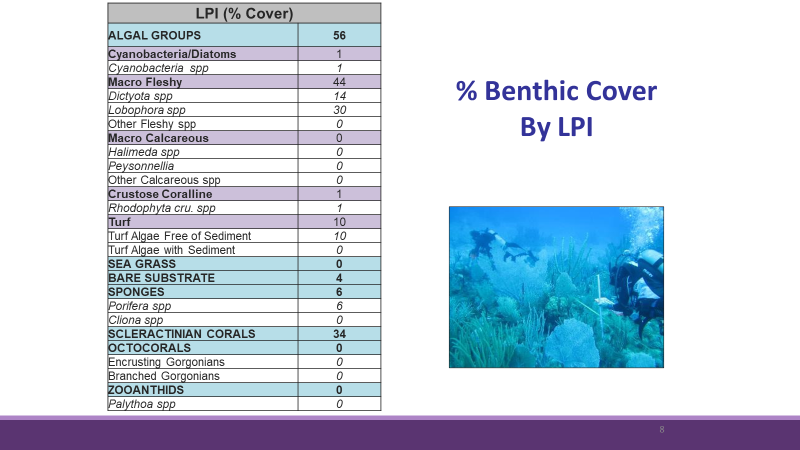


Figure G2: LPI homework page that contains LPI information given to expert panel to provide context for reef condition to decide their BCG level ratings.


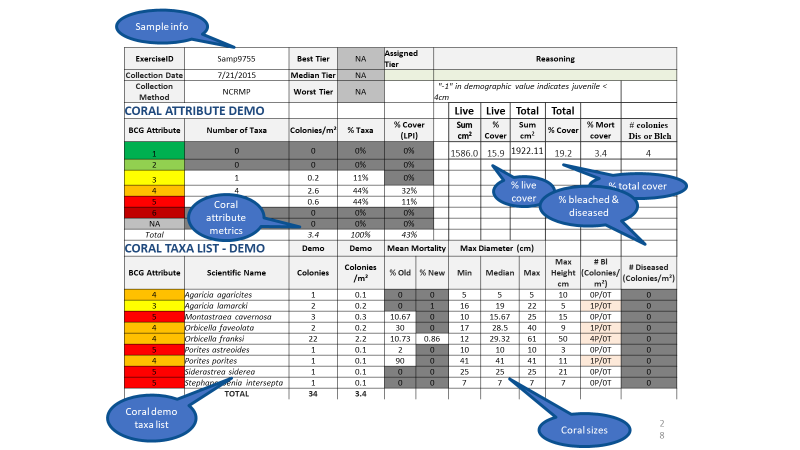


Figure G3: Site attributes on homework page given with the metrics derived from DEMO method that were used by the expert panel to decide their BCG level ratings.


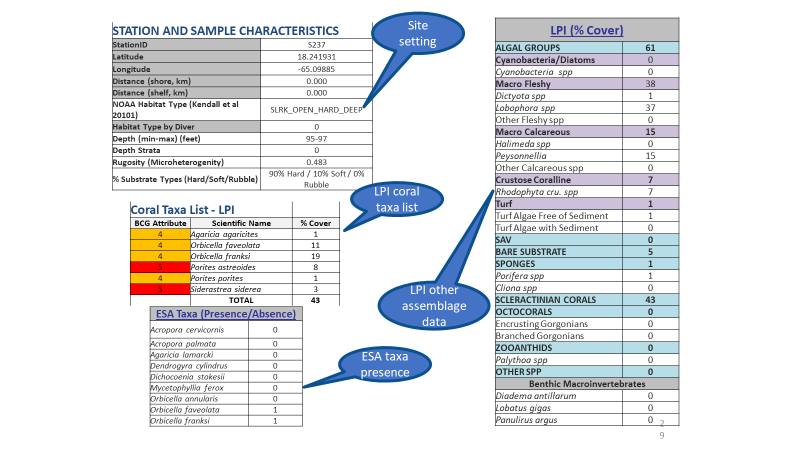


Figure G4: Site attributes on homework page given to experts with the metrics derived from LPI method that were used by the expert panel to decide their BCG level ratings.
