# Supplementary material for: A biological condition gradient for Caribbean coral reefs: Part II. Numeric rules using sessile benthic organisms
Source: Ecol Indic. Author manuscript; Available in PMC 2022 May 4. (PMC9067392; doi:10.1016/j.ecolind.2022.108576)
Supplement: Supplementary data 3. [file NIHMS1794197-supplement-Supplementary_data_3_.docx]

**Supplemental Information C**

**Expert panel members and affiliations for numeric model development**

Dr. David Ballantine

Department of Botany

Smithsonian National Museum of Natural History

Washington, DC, 20560

Mr. Randy Clark

National Oceanic and Atmospheric Administration

National Center for Coastal Ocean Science, Marine Spatial Ecology Division

Stennis Space Center, MS 39529

Dr. David Cuevas

US Environmental Protection Agency, Region 2

Caribbean Environmental Protection Division

Guaynabo, PR 00968

Mr. Ernesto Diaz

PR Department of Natural and Environment Resources (DNER)

San Juan, PR 00936

Dr. William Fisher

US Environmental Protection Agency

Office of Research and Development, Gulf Ecosystem Measurement and Modeling Division (GEMMD)

Gulf Breeze, FL 32561

Dr. Chris Jeffrey

CSS-Dynamac, National Oceanic and Atmospheric Administration

Fairfax, VA 22030

Dr. Ángel R Meléndez-Aguilar

Water Quality Area

Puerto Rico Environmental Quality Board

San Juan, PR 00910

Ms. Tania M. Metz

Puerto Rico Coral Reef Program

PR DNER

San Juan, PR 00915

Dr. Caroline S Rogers

United States Geological Survey

Wetland and Aquatic Research Center

Caribbean Field Station

St. John, USVI 00830

Dr. Tyler Smith

University of the Virgin Islands
St. Thomas, VI 00802

Dr. Alina Szmant

University of North Carolina, Wilmington

Center for Marine Science

Wilmington, NC 28409

Ms. Brandi Todd

National Oceanic and Atmospheric Administration

Office of Research and Response

New Orleans, LA 70130

Dr. Hector Ruiz Torres

HJR Reefscaping

Hormigueros, PR 00660

Dr. Brian K. Walker

National Coral Reef Institute

Nova Southeastern University

Dania Beach, FL 33004

Dr. Ernesto Weil

University of Puerto Rico

Mayagüez Campus

Lajas, PR 0066
