# Supplementary material for: A biological condition gradient for Caribbean coral reefs: Part II. Numeric rules using sessile benthic organisms
Source: Ecol Indic. Author manuscript; Available in PMC 2022 May 4. (PMC9067392; doi:10.1016/j.ecolind.2022.108576)
Supplement: Supplementary data 2. [file NIHMS1794197-supplement-Supplementary_data_2_.docx]

**Supplemental Information B**

**Coral metric definitions and formulas used in numeric model**

For each coral colony within the 10 x 1 m^2^ transect, the maximum height (h_cm), maximum diameter (max_d_cm) and diameter perpendicular to the maximum diameter (p_d_cm), species name, and percent tissue (%LT=live coral visually assessed in 10% increments) were recorded.

The coral demographic metrics used for BCG development (adapted from Santavy et al. 2012; Bradley et al. 2014) were Colony Surface Area (CSA), Live tissue area on Colony Surface Area (LCSA), and Live Colony Surface Area 2D planar view (LCSA_2D). The CSA was the total surface area (cm^2^) of a single colony, which includes both living tissue covering the skeleton and dead portions on the three-dimensional skeletal surface, such that:

CSA = πr^2^ M *(1)*

where, r = [h_cm+ (max_d_cm/2)] /2 *(2)*

The variables used to calculate r were: h_cm=maximum colony height (cm), max_d_cm=maximum colony diameter (cm), and M = morphological conversion factor. In general, morphological types and relative values included flat (M=1), hemisphere (M=2), overlapping plates and lobes (M=3), and branched (M=4) colonies (See Supplemental Table B1). The LCSA was the total surface area (cm^2^) of a single colony, which included only the living tissue that covered the skeletal surface and was calculated as:

LCSA= CSA (%LT/100) *(3)*

Where %LT was the estimated percent of colony surface area that contained live tissue. The LCSA_2D was an estimated value of the total planar colony surface area (cm^2^) of living tissue on a single coral colony as though it were viewed only from directly above the colony and was estimated as:

LCSA_2D = π [2r (cm)/2]^2^ * (%LT/100) *(4)*

This calculation assumes equal distribution of living tissue on a colony, which was initially recorded.

| Table B1: Morphological conversion factors for Western Atlantic and Caribbean scleractinian species used to calculate colony surface area (Santavy et al. 2012). | |
| --- | --- |
| Genus and Species | Conversion Factor |
| *Acropora cervicornis* | 4 |
| *Acropora palmata* | 4 |
| *Acropora prolifera* | 4 |
| *Agaricia agaricites* | 1 |
| *Agaricia fragilis* | 1 |
| *Agaricia humilis* | 1 |
| *Agaricia lamarcki* | 1 |
| *Agaricia tenuifolia* | 3 |
| *Cladocora arbuscula* | 2 |
| *Colpophyllia natans* | 2 |
| *Dendrogyra cylindrus* | 3 |
| *Dichocoenia stokesii* | 2 |
| *Diploria labyrinthiformis* | 2 |
| *Eusmilia fastigiata* | 3 |
| *Favia fragum* | 2 |
| *Helioseris cucullata* | 1 |
| *Isophyllia rigida* | 2 |
| *Isophyllia sinuosa* | 2 |
| *Madracis decactis* | 3 |
| *Madracis formosa* | 3 |
| *Madracis aurentenea* | 3 |
| *Madracis pharensis* | 1 |
| *Manicina areolata* | 2 |
| *Meandrina meandrites* | 2 |
| *Montastraea cavernosa* | 2 |
| *Mussa angulosa* | 2 |
| *Mycetophyllia aliciae* | 1 |
| *Mycetophyllia danaana* | 1 |
| *Mycetophyllia ferox* | 1 |
| *Mycetophyllia lamarckiana* | 1 |
| *Oculina varicosa* | 3 |
| *Orbicella annularis* | 3 |
| *Orbicella faveolata* | 2 |
| *Orbicella franksi* | 2 |
| *Porites astreoides* | 2 |
| *Porites colonensis* | 1 |
| *Porites divaricata* | 3 |
| *Porites furcata* | 3 |
| *Porites porites* | 3 |
| *Pseudodiploria clivosa* | 2 |
| *Pseudodiploria strigosa* | 2 |
| *Siderastrea siderea* | 2 |
| *Solenastrea bournoni* | 2 |
| *Solenastrea hyades* | 3 |
| *Stephanocoenia intersepta* | 2 |

**References**

Bradley, P., Santavy, D.L., Gerritsen, J. 2014. Workshop on Biological Integrity of Coral Reefs, August 21-22, 2012, Caribbean Coral Reef Institute, Isla Magueyes, La Parguera, Puerto Rico. US Environmental Protection Agency, Office of Research and Development, Atlantic Ecology Division, Narragansett, RI. EPA/600/R-13/350.

Santavy, D.L., Fisher, W.S., Campbell, J.G., Quarles R.L. 2012. Field manual for coral reef assessments. U.S. Environmental Protection Agency, Office of Research and Development, Gulf Ecology Division, Gulf Breeze, FL. EPA/ 600/R-12/029. April 2012.
