# Supplementary material for: A biological condition gradient for Caribbean coral reefs: Part II. Numeric rules using sessile benthic organisms
Source: Ecol Indic. Author manuscript; Available in PMC 2022 May 4. (PMC9067392; doi:10.1016/j.ecolind.2022.108576)
Supplement: Supplementary data 1. [file NIHMS1794197-supplement-Supplementary_data_1_.docx]

**Supplemental Information A**

**NOAA National Coral Reef Monitoring Program (NCRMP) protocols and survey designs**

NOAA’s National Coral Reef Monitoring Program (NCRMP) has been conducting nationally coordinated, continuous, and standardized coastal ocean assessments to develop biological, climate, and socioeconomic status and trends indicators for priority U.S. coral reef areas since 2013. Surveys are conducted in Florida, Puerto Rico, U.S. Virgin Islands (USVI), Flower Garden Banks, Hawaii, Guam, American Samoa, Commonwealth of the Northern Marianas, and the Pacific Remote Island Areas. The NCRMP is a stratified random sampling design, where the sampling domain for each region (e.g., Puerto Rico, USVI, Flower Garden Banks, Florida) is partitioned by habitat type and depth strata (0-30 m), habitat location/reef types (e.g., along-shelf position) and management zone. NCRMP supplements local monitoring efforts by providing regional scale data on reef fishes and the coral reef benthic assemblages. In 2013, NOAA implemented the first year of its National Coral Reef Monitoring Program (NCRMP) in USVI and released NCRMP guidance for the Caribbean in 2014 (NOAA 2014), and regularly thereafter. NOAA and their partners (University of Virgin Islands, US National Park Service, University of Miami) monitored coral community structure, fish, and benthic cover estimates for ecologically important cover types/groups.

**NOAA NCRMP Bioassessment Design and Survey**

The NOAA NCRMP survey design focused on determining the status and trends of coral reef condition; however, different metrics and benthic assemblages were chosen to achieve different goals at several smaller spatial scales than those employed in the EPA surveys. The randomized sampling design was stratified by depth (e.g., shallow ≤ 12 m; > 12 m- 30 m deep); reef geographic zone (e.g., back reef, fore reef, bank/shelf, etc.) and habitat type based on geomorphological structure (e.g., spur and groove, pavement, patch reef, aggregate reef) (Costa et al. 2013; Zitello et al. 2009); and management zone (e.g., MPA, no‐take area, etc.) (NOAA Coral Program 2014). Selection of the sampling frames and sites depended on the quality and extent of available maps for the bathymetry and habitat descriptions of coral reefs that was verified from partners with local resource knowledge from the respective jurisdictions and sub-jurisdictions. NCRMP targeted sessile benthic assemblages and fish communities in a stratified random sampling design.

Bioassessment data from NCRMP Puerto Rico and the USVI surveys were collected in 2013 – 2015 and were used for developing the numeric BCG benthic rules. The measurements not in the EPA database identified by the expert panel were recorded in the NCRMP protocols for coral and other benthos. Much of the non-coral data were not obtained using the Line-Point Intercept method (LPI), NOAA did not include sponges and gorgonians in their DEMO surveys, NOAA used a microheterogeneity approach for reef rugosity. The expert panel recognized natural differences in benthic reef assemblages inhabiting shallow and deep sites. The deepest sites in the data set were approximately 30 m deep, which they determined as the maximum practical depth for routine underwater monitoring. Within this depth range, the BCG expert panel suggested that differences in reef structure occurred at approximately 12 m depth, from gradual and general differences in light penetration and wave action. There was an effort to concentrate on shallow reef sites (<12 m). However, a fuller range of conditions was only found among NCRMP data when both shallow and deep sites were included.

**Line-Point Intercept (LPI) Method**

NCRMP employed the LPI method to estimate percent planar coverage of ecologically important benthic assemblages (macroalgae, turf algae, crustose coralline algae, corals, sponges, sand/sediment, etc.) (Figure A1). Points along a 25 m transect were used to quantify each of the benthic organism or substrate type lying every 20 cm beneath the tape, for a total of 100 points to tally substrates and biota. Along the 25 m transect, divers reported the presence or absence survey for US Endangered Species Act (ESA) listed species and selected macroinvertebrates). In the 2013, surveys included the threatened coral species *Acropora cervicornis* and *Acropora palmata*. Beginning in 2014, five additional newly listed Caribbean coral species were included: *Dendrogyra cylindrus, Orbicella annularis, Orbicella faveolata, Orbicella franksi*, and *Mycetophyllia ferox* (NOAA 2012a, b). The density of *Aligers gigas* (queen conch), *Panulirus argus* (spiny lobster), Scyllaridae (slipper lobster), and *Diadema antillarum* (sea urchins) were recorded (Santavy et al. 2012). Some underwater photographs were taken along the 25 m transect. Only summary statistics for percent cover for taxa richness were used for all other assemblages except scleractinian corals., where each species percent cover was recorded.

**Demographic Method Survey (DEMO)**

NCRMP DEMO surveys were conducted at a subset of LPI sample sites (2013: 220 DEMO sites/283 LPI sites; 2014: 111 DEMO sites/230 LPI sites; 2015: 139 DEMO sites/239 LPI sites). Divers swam along a 10 m x 1 m belt transect over hard-bottom and coral reef habitats, recording information on coral species composition, density, size, abundance, and specific parameters of condition (% live vs. dead and type of bleaching) for non-juvenile scleractinian corals (> 4 cm maximum diameter), and of overall species diversity (all corals (Figure A1)). From the species, size, and mortality measures obtained in the DEMO surveys, coral surface area (CSA) and live CSA (LCSA) were calculated in 2 and 3 dimensions.

**Rugosity Microheterogeneity Measure**

The NCRMP 2013-2015 surveys used a microheterogeneity measure to estimate reef rugosity, which was the difference between the lowest and highest points in quadrats along the transect, averaged for all quadrats at a site (Brandt et al. 2009). Fine scale rugosity measurements were made to estimate the reef surface topography in the NCRMP assessments. Hard bottom relief was measured at 24 locations along the 25 m LPI transect and recorded as centimeters binned into six height classes (<0.2 m, 0.2-<0.5 m, 0.5-<1.0, 1.0-<1.5 m, 1.5-<2.0 m, >2 m). Using the frequencies from each transect a single rugosity index was calculated. The frequency of each height class used as the midpoint of each height class (lowest to highest: 0.1, 0.35, 0.75, 1.25, 1.75, actual height if >2 m) multiplied by the number of observations in that height class. If the height was >2 m, the maximum vertical height was the multiplier. Finally, the sum of the products from all height classes was divided by the total number of observations (24) to obtain the microheterogeneity rugosity value (MRV) (NOAA Coral Program 2014; NOAA NCRMP 2014 Puerto Rico 2014; NOAA NCRMP 2013 USVI 2013). The maximum and minimum transect depths were noted.

Figure A1. Diagram of NCRMP surveys. LPI and DEMO surveys conducted as the divers swam away from the transect origin. Other invertebrate assemblages and topographic complexity were surveyed. (NOAA 2014)

**References**

Brandt, M.E., Zurcher, N., Acosta, A., Ault, J.S., Bohnsack, J.A., Feeley, M.W., Harper, D.E., Hunt, J.H., Kellison, T., McClellan, D.B., Patterson, M.E., Smith, S.G. 2009. A cooperative multi‐agency reef fish monitoring protocol for the Florida Keys coral reef ecosystem. Natural Resource Report NPS/SFCN/NRR‐2009/150, Fort Collins, Colorado, National Park Service.

Costa, B.M., Kendall, M.S., Edwards, K., Kagesten, G., Battista, T.A. 2013. Benthic Habitats of Fish Bay, Coral Bay, and the St. Thomas East End Reserve. NOAA Technical Memorandum NOS NCCOS 175.

NOAA. 2012a. Draft Management Report for 82 Corals Status Review under the Endangered Species Act: Existing Regulatory Mechanisms (per Endangered Species Act § 4(a)(1)(D), 16 U.S.C. § 1533(a)(1)(D)) and Conservation Efforts (per Endangered Species Act § 4(b)(1)(A), 16 U.S.C. § 1533(b)(1)(A)). Pacific Islands Regional Office, National Marine Fisheries Service, 233 pp.

NOAA. 2012b. Endangered and threatened wildlife and plants—Proposed listing determinations for 82 reef-building coral species—Proposed reclassification of *Acropora palmata* and *Acropora cervicornis* from threatened to endangered. Federal Register 77(236): 73221–73261.

NOAA. 2014. NOAA’s State of the Coast website. http://stateofthecoast.noaa.gov/ (last accessed October 2021)

NOAA Coral Program. 2014. National Coral Reef Monitoring Plan. Silver Spring, MD, NOAA. Coral Reef Conservation Program. 40 pp.

NOAA NCRMP 2014 Puerto Rico. 2014. NCRMP assessment data: Assessment of coral reef benthic communities in Puerto Rico; https://data.nodc.noaa.gov/cgi-bin/iso?id=gov.noaa.nodc:NCRMP-Benthic-PR (last accessed October 2021).

NOAA NCRMP 2013 USVI. 2013. NCRMP assessment data: Assessment of coral reef benthic communities in the U.S. Virgin Islands; https://data.nodc.noaa.gov/cgi-bin/iso?id=gov.noaa.nodc:NCRMP-Benthic-USVI, (last accessed October 2021).

Santavy, D.L., Fisher, W.S., Campbell, J.G., Quarles R.L., 2012. Field manual for coral reef assessments. U.S. Environmental Protection Agency, Office of Research and Development, Gulf Ecology Division, Gulf Breeze, FL. EPA/ 600/R-12/029. April 2012.

Zitello, A.G., L.J. Bauer, T.A. Battista, P.W. Mueller, M.S. Kendall, Monaco, M.E., 2009. Shallow-Water Benthic Habitats of St. John, U.S. Virgin Islands. NOAA Technical Memorandum NOS NCCOS 96. Silver Spring, MD.
